# The peroxisome proliferator-activated receptor agonist rosiglitazone specifically represses tumour metastatic potential in chromatin inaccessibility-mediated FABP4-deficient gastric cancer

**DOI:** 10.7150/thno.66814

**Published:** 2022-01-24

**Authors:** Qi-Yue Chen, Xiao-Bo Huang, Ya-Jun Zhao, Hua-Gen Wang, Jia-Bin Wang, Li-Chao Liu, Ling-Qian Wang, Qing Zhong, Jian-Wei Xie, Jian-Xian Lin, Jun Lu, Long-Long Cao, Mi Lin, Ru-Hong Tu, Chao-Hui Zheng, Ping Li, Chang-Ming Huang

**Affiliations:** 1 Department of Gastric Surgery, Fujian Medical University Union Hospital, Fuzhou 350001, Fujian, P. R. China; 2 Key Laboratory of Ministry of Education of Gastrointestinal Cancer, Fujian Medical University, Fuzhou 350001, Fujian, P. R. China; 3 Fujian Key Laboratory of Tumor Microbiology, Fujian Medical University, Fuzhou 350001, Fujian, P. R. China; 4 The First Affiliated Hospital of USTC, Division of Life Sciences and Medicine, University of Science and Technology of China, Hefei 230001, Anhui, P. R. China

**Keywords:** Gastric cancer, FABP4, PPAR-γ, CADM3, Rosiglitazone

## Abstract

**Background:** Efforts to prevent recurrence in gastric cancer (GC) patients are limited by current incomplete understanding of the pathological mechanisms. The present study aimed to identify novel tumour metastasis-associated genes and investigate potential value of these genes in clinical diagnosis and therapy.

**Methods:** RNA sequencing was performed to identify differentially expressed genes related to GC metastasis. The expression and prognostic significance of fatty acid binding protein 4 (FABP4) were evaluated in two independent cohorts of GC patients. Chromatin immunoprecipitation sequencing, diverse mouse models and assays for transposase-accessible chromatin with high-throughput sequencing were used to investigate the roles and mechanisms of action of FABP4.

**Results:** The results of the present multicentre study confirmed an association between a decrease in the expression of FABP4 and poor outcomes in GC patients. FABP4 inhibited GC metastasis but did not influence tumour growth *in vitro* and *in vivo*. Mechanistically, FABP4 binding with peroxisome proliferator-activated receptor γ (PPAR-γ) facilitated the translocation of PPAR-γ to the nucleus. FABP4 depletion suppressed PPAR-γ-mediated transcription of cell adhesion molecule 3 (CADM3), which preferentially governed GC metastasis. Notably, the PPAR-γ agonist rosiglitazone reversed the metastatic properties of FABP4-deficient GC cells *in vitro* and demonstrated viable therapeutic potential in multiple mouse models. For GC patients with diabetes, low FABP4 portends better prognosis than high FABP4 after receipt of rosiglitazone treatment. Additionally, chromatin inaccessibility induced by HDAC1 reduced FABP4 expression at the epigenetic level.

**Conclusions:** Our findings suggest that chromatin inaccessibility orchestrates a reduction in FABP4 expression, which inhibits CADM3 transcription via PPAR-γ, thereby resulting in GC metastasis. The antidiabetic drug rosiglitazone restores PPAR-γ/CADM3 activation in FABP4-deficient GC and thus has promising therapeutic potential.

## Introduction

Gastric cancer (GC) is a highly aggressive disease that is the third leading cause of cancer-related mortality worldwide 1. Despite improvements in radical resection with adjuvant chemotherapy, metastasis and recurrence remain the main lethal factors in advanced GC patients 2, 3. In particular, 20%-30% of GC cases involve peritoneal metastasis (PM), with a median survival time of approximately 1 year 4, 5. A deeper understanding of GC metastasis is important for identification of the drivers and corresponding effective treatments of this disease.

Numerous biomarkers have been proposed to explain the acquisition of metastatic competence by GC cells 6-8; however, the studies mostly focused on differential expression of these insufficiently specific molecules between tumour and adjacent tissues. Very few studies reported targeted analysis of GC metastasis or recurrence, which limits the design of effective treatments. Initial studies demonstrated that fatty acid binding protein 4 (FABP4) mediates multiple physiological processes, including insulin sensitivity, attenuated atherosclerosis and inflammatory response 9-11. Recent studies demonstrated that pro- or antitumour roles of FABP4 are dependent on cancer type 12, 13. For example, FABP4 facilitates the stemness and aggressiveness of tumour cells via the IL-6/STAT3/ALDH1 axis in obesity-associated breast cancer 14. And, a small-molecule inhibitor of FABP4 (BMS309403) efficiently blocked early metastasis and reduced tumour burden in an orthotropic mouse model of ovarian cancer 15. While in contrast, FABP4 exhibited an antitumour effect on hepatocellular carcinoma cells and was associated with favourable prognosis of patients 16. To date, FABP4 is rarely reported in GC and its role as well as mechanism in GC has not been fully elucidated.

Herein, we identified FABP4 as a metastasis-specific gene through robust bioinformatics analysis. We highlighted the biological significance of FABP4 in GC and demonstrated a previously unrecognized molecular mechanism underlying GC metastasis. Moreover, we identified rosiglitazone as an effective mechanism-based intervention against FABP4 in GC, suggesting that FABP4 was a promising target for novel diagnostic and therapeutic strategies.

## Methods

### Patients and tissue samples

Tissue samples were obtained from patients diagnosed with GC at Fujian Medical University Union Hospital (FMUUH) (Fuzhou, Fujian, China) and the First Affiliated Hospital of University of Science and Technology of China (USTC, Hefei, Anhui, China). Patient enrolment and study overview are presented in **Figure S1A**. Twenty fresh samples of GC tissues were obtained at FMUUH in 2018 from patients with advanced GC with or without peritoneal metastasis (PM) and no other distant metastases; the patients did not have combined malignancies and were not previously treated with chemotherapy or radiotherapy. Suitable specimens were analysed by RNA sequencing (RNA-seq). A total of 289 fresh samples of GC and adjacent normal tissues were collected randomly between 2017 and 2018 at FMUUH to detect the expression of FABP4.

A total of 352 paraffin-embedded samples of GC and adjacent normal tissues were collected from 2010 to 2015 at FMUUH and used for immunohistochemistry (IHC). A total of 123 paraffin-embedded samples of GC tissues were obtained at the First Affiliated Hospital of USTC between 2013 and 2014 and were used for validation of clinical prognostic and correlation analysis. The inclusion criteria were as follows: (1) histological identification of GC; (2) absence of combined malignancies and distant metastases; (3) availability of complete follow-up data; and (4) initial or updated tumour stage classification performed according to the 7^th^ edition of The American Joint Committee on Cancer (AJCC) cancer staging manual 17. Recurrence was defined as the presence of a biopsy-documented tumour or imaging features and was categorized according to location. The present study was approved by the ethics committee of FMUUHH (no. 2020KY0150) and the First Affiliated Hospital of USTC (no. 2020-WCK-01), and written consent was obtained from all enrolled patients.

### Cell lines

Human GC lines (AGS, BGC-823, and MGC-803) were grown in RPMI-1640 medium (Gibco, Carlsbad, CA, USA) or F-12K medium (Gibco, Carlsbad, CA, USA) containing 10% foetal bovine serum (Gibco, Carlsbad, CA, USA) and maintained in a 5% CO_2_ incubator at 37°C. AGS cells were obtained from Cellcook Biotech Co., Ltd. (Guangzhou, Guangdong, China). BGC-823 cells were purchased from GeneChem Corporation (Shanghai, China). MGC-803 cells were obtained from the Cell Line Bank, Chinese Academy of Sciences (Shanghai, China). All cell lines were mycoplasma-free and were authenticated using short tandem repeat (STR) profiling analysis.

### Animal models

Male BALB/c nude mice (4-5-weeks-old) used in the present study were purchased from Beijing Vital River Laboratory Animal Technology Co., Ltd. (Beijing, China). All experiments were performed according to the guidelines of the Animal Protection Committee of Fujian Medical University (Fuzhou, Fujian, China) and approved by the Ethics Committee of Fujian Medical University/Laboratory Animal Center (Fuzhou, Fujian, China).

To evaluate the effect of FABP4 on GC growth, the right axillary fossa of mice was transplanted subcutaneously with a total of 2×10^6^ stably transfected MGC-803 cells. Tumour volume was measured every week using calipers and calculated according to the following equation: V = (L × W^2^)/2 (V, tumour volume; L, length; and W, width). Three-five weeks after the injection, the tumours were isolated from the mice and weighed.

Two metastatic models were generated to detect the effect of FABP4 on GC metastasis. To generate a lung metastasis model, 2×10^6^ stably transfected BGC-823 cells diluted in 200 μL of PBS were injected into the tail veins of mice. Four weeks later, the lungs were isolated from mice and used to assess the numbers of metastatic loci. To generate a liver metastasis model, 8×10^5^ stably transfected BGC-823 or MGC-803 cells diluted in 80 μL PBS were injected into the spleens of mice. Four or seven weeks later, the livers were dissected to evaluate the metastatic ability of BGC-823 and MGC-803 cells, respectively. We also generated these two metastatic models using luciferase-labelled BGC-823 cells to investigate the effect of rosiglitazone on the metastatic ability of FABP4-deficient GC cells. One week after the transplantation, the mice were treated daily with DMSO (Solarbio, Beijing, China) or rosiglitazone (10 mg/kg or 20 mg/kg; MedChemExpress, Shanghai, China) via intraperitoneal injection.

### Transcriptomic RNA sequencing

Total RNA was extracted with TRIzol reagent (Invitrogen, Carlsbad, CA, USA). Transcriptomic RNA-seq was performed at KangChen Bio-tech Inc. (Shanghai, China). Briefly, a total of 1~2 μg of RNA per sample was used to construct a cDNA library. The quality of the constructed library was evaluated using an Agilent 2100 Bioanalyzer (Agilent Technologies, Santa Clara, CA, USA). Library sequencing was performed on the Illumina HiSeq 4000 platform (Illumina, San Diego, California, USA).

### Public datasets

The TCGA-STAD (The Cancer Genome Atlas stomach adenocarcinoma) and GSE15459 datasets were used in the present study. The TCGA-STAD dataset was downloaded from the Genomic Data Commons (GDC) data portal (http://portal.gdc.cancer.gov/), and the GSE15459 dataset was downloaded from the Gene Expression Omnibus (GEO) database (http://www.ncbi.nlm.nih.gov/geo/). cBioPortal for Cancer Genomics (http://www.cbioportal.org/) was used to analyse FABP4 alterations in the TCGA dataset, including CNV, mutation, and DNA methylation. MethPrimer (http://www.urogene.org/) was used to predict the CpG islands within the FABP4 promoter. ATAC-seq results of human GC tissues were acquired from GDC (http://gdc.cancer.gov/about-data/publications/ATACseq-AWG).

### Chromatin immunoprecipitation sequencing analysis

ChIP-seq analysis was performed at Igenebook Biotechnology Co., Ltd. (Wuhan, China) using the stably transfected cells. Briefly, the cells were cross-linked with formaldehyde, lysed, and sonicated to produce chromatin fragments of 200~500 bp, which were then immunoprecipitated with anti-Flag (5 µg; MAB3118; Millipore, Billerica, MA, USA). Normal rabbit IgG (5 μg; 2729; Cell Signaling Technology, Danvers, MA, USA) was included as a nonspecific control.

### Immunohistochemistry

Tissue specimens were fixed with formalin, embedded in paraffin and serially sectioned at a thickness of 4 μm. The sections were deparaffinized with dimethylbenzene and rehydrated in a graded series of ethanol. Antigen retrieval was performed in sodium citrate buffer (MXB Biotechnologies, Fuzhou, Fujian, China), and endogenous peroxidase activity was blocked by hydrogen peroxide (MXB Biotechnologies, Fuzhou, Fujian, China). The sections were then blocked with 10% goat serum (Gibco, Carlsbad, CA, USA) and incubated with primary antibodies at 4°C overnight. The following primary antibodies were used: rabbit anti-FABP4 (1:1,000; ab13979; Abcam, Cambridge, MA, USA), rabbit anti-CADM3 (1:200; 343894; USBiological, Swampscott, MA, USA) and rabbit anti-HDAC1 (1:4,000; ab19845; Abcam, Cambridge, MA, USA). Subsequently, the sections were incubated with a secondary antibody (ZhongShan Biotechnology, Beijing, China) at room temperature for 30 min. The signal was developed in diaminobenzidine (DAB) solution (ZhongShan Biotechnology, Beijing, China), and the slides were counterstained with haematoxylin (Beyotime, Shanghai, China). Finally, the slides were scanned and analysed by Motic Easyscanner (Motic, Hong Kong, China). The IHC scores ranging from 0 to 9 were calculated by multiplying the intensity and heterogeneity scores, and the patients were categorized according to the IHC scores into low (0, 1, 2 and 3) and high (4, 6 and 9) groups.

### RNA extraction and real-time PCR

Total RNA was isolated from the cell line and tissue samples using TRIzol reagent (Invitrogen, Carlsbad, CA, USA). cDNA was prepared from 800 ng total RNA using PrimeScript RT Master Mix (Takara, Dalian, China) in accordance with the manufacturers' instructions. The expression of mRNAs was measured using SYBR Green PCR Master mix (Takara, Dalian, China) on a real-time PCR instrument (Bio-Rad, Hercules, CA, USA). The data were analysed using the 2^-ΔΔCT^ method with GAPDH as a control. The primers used in the present study are listed in **Table S1**.

### Protein isolation and western blotting

The cells were extracted into RIPA lysis buffer (Beyotime, Beijing, China) containing a protease inhibitor cocktail (Sigma-Aldrich, Saint Louis, MO, USA), and the lysates were centrifuged at 12,000 rpm (4°C for 15 min). Protein concentration was quantified using a BCA protein assay kit (Thermo Scientific, Waltham, MA, USA). The following primary antibodies were used: rabbit anti-FABP4 (1:500; ab13979; Abcam, Cambridge, MA, USA), rabbit anti-FABP5 (1:1,000; bs-2370R; Bioss, Bejing, China), rabbit anti-PPAR-γ (1:1,000; 2443S; Cell Signaling Technology, Danvers, MA, USA) and rabbit anti-CADM2 (1:1,000; bs-8246R; Bioss, Bejing, China), rabbit anti-CADM3 (1:200; 343894; USBiological, Swampscott, MA, USA), rabbit anti-CADM4 (1:1,000; bs-5997R; Bioss, Bejing, China), rabbit anti-HDAC1 (1:1,000; ab19845; Abcam, Cambridge, MA, USA), rabbit anti-HDAC2 (1:1,000; bs1813R; Bioss, Bejing, China), rabbit anti-HDAC6 (1:1,000; bs-2811R; Bioss, Bejing, China), rabbit anti-HDAC8 (1:1,000; bsm-52088R; Bioss, Bejing, China), rabbit anti-GAPDH (1:1,000; ab9485; Abcam, Cambridge, MA, USA) were used as a control. Signal detection was performed using an ImageQuant LAS 4000 mini instrument (GE Healthcare, Piscataway, NJ, USA).

### RNA interference

The small interfering RNAs (siRNAs) targeting PPAR-γ and CADM3 were synthesized by GenePharma (Shanghai, China). Transient transfection with siRNAs was performed using lipofectamine 3000 (Thermo Scientific, Waltham, MA, USA) according to the established protocols. Briefly, the cells were seeded in 6-well plates (5 × 10^4^ cells) and then infected with lentiviral particles for 12 h. Puromycin (Sigma-Aldrich, Saint Louis, MO, USA) was used to select stable clones of cultured cells for at least one week. Finally, stably transfected cell lines were harvested for subsequent experiments.

### Cell proliferation, migration, invasion, and adhesion assays and cell apoptosis analysis

Cell proliferation assays were performed using a Cell Counting Kit-8 (CCK-8; Dojindo, Kumamoto, Japan). Briefly, 1 × 10^3^ cells were plated per well of 96-well plates and cultured for 24 h. Ten microlitres of CCK-8 solution was added to the wells and incubated for indicated time intervals. Then, the absorbance was examined at 450 nm using a microplate reader (Bio-Rad, Hercules, CA, USA). Transwell assays were performed to detect the migration and invasion of GC cells using Transwell chambers (BD Bioscience, San Diego, CA, USA). A total of 1×10^5^ cells in 200 μl of serum-free medium were seeded on top of the Transwell inserts, and 500 μl of complete medium containing 10% FBS was added to the lower chamber. After 12~24 h, the cells that migrated to or invaded the bottom chamber were stained with 0.5% crystal violet. Three randomly selected fields of view were analysed for each chamber (magnification, × 200). Cell aggregation was imaged under 100 × magnification, and the volume of the aggregates was calculated according to the following equation: V = a × b^2^ × π/6 cm^3^ (a, the longest diameter of the aggregates; b, the shortest diameter of the aggregates). The volume of each cell aggregates was calculated as the average volume of at least 6 cell aggregates. Apoptosis rates of the cells were analysed by flow cytometry using a FITC Annexin V apoptosis detection kit I (BD Bioscience, San Diego, CA, USA). The cells were stained and filtered through a 70 μm cell strainer immediately prior to flow cytometry performed using a FACSVerse flow cytometer (BD Biosciences, San Jose, CA).

### Co-immunoprecipitation assay

Cells were washed with PBS and lysed in IP lysis buffer (Beyotime, Shanghai, China). The lysates were incubated with rabbit anti-FLAG (1:50; 14793S; Cell Signaling Technology, Danvers, MA, USA) and anti-PPAR-γ (1:50; 2443S; Cell Signaling Technology, Danvers, MA, USA) antibodies overnight at 4°C. Protein A/G PLUS-agarose beads (Santa Cruz Biotechnology, CA, USA) were added to the lysates and incubated for another 2~4 h at 4°C. The immunoprecipitates were washed 5 times in IP lysis buffer, boiled for 5 min in 40 µL of 1× SDS buffer and then assayed by western blotting.

### Immunofluorescence assay

The cells grown on glass bottom dishes (Cellvis, Mountain View, CA, USA) were washed with PBS and fixed with 4% paraformaldehyde. The cells were blocked with Triton X-100 (Beyotime, Shanghai, China). Subsequently, the cells were incubated with rabbit anti-PPAR-γ (1:100; 2435S; Cell Signaling Technology, Danvers, MA, USA) antibodies overnight at 4°C. Additionally, all samples were treated with 4′,6-diamidino-2-phenylindole (DAPI) (Solarbio, Beijing, China) to stain the nuclei. Finally, a TCS SP8 confocal microscope (Leica, Wetzlar, Germany) was used for imaging.

### Cycloheximide (CHX) chase assay and ubiquitination assay

For the CHX chase assay, the cells were treated with CHX (25 μg/ml; MedChemExpress, Shanghai, China) for indicated time intervals, and cell lysates were analysed by western blotting. For the ubiquitination assay, the cells were treated with the proteasome inhibitor MG132 (10 μM; MedChemExpress, Shanghai, China) for 6 h, and cell lysates were analysed by western blotting.

### Dual-luciferase reporter assay

Cells seeded in 24-well plates were cotransfected with the CADM3 promoter, PPAR-γ, FABP4 and control plasmids. Briefly, the transcription factor-binding sites of the CADM3 promoter region were predicted using UCSC Genome Browser (http://genome.ucsc.edu/) and the JASPAR database (http://jaspar.genereg.net/). After 48 h of culture, the luciferase activities of the cells were measured using the double-luciferase reporter assay kit (TransGen Biotech, Beijing, China) according to the manufacturer's protocols. Renilla luciferase activity was used to normalize for transfection efficiency. The experiments were performed in triplicate.

### Statistical analysis

Statistical analysis was performed using SPSS software version 16.0 (IBM Corporation Armonk, New York, USA) and GraphPad Prism version 8.0 (GraphPad Software, San Diego, California, USA). The data are presented as the mean ± SD. Parametric Student's t-test or nonparametric Mann-Whitney test were used for comparison of two groups. Chi-squared test was used to examine associations between two categorical variables. Spearman correlation test was used for correlation analysis. Kaplan-Meier analysis was used to compare survival distributions of two groups based on the log-rank test. Prognostic factors were evaluated by univariate and multivariate Cox proportional hazards models. *P* < 0.05 was considered statistically significant.

## Results

### Downregulation of FABP4 is associated with decreased survival and an increase in the recurrence rate in GC patients

**Figure 1:**
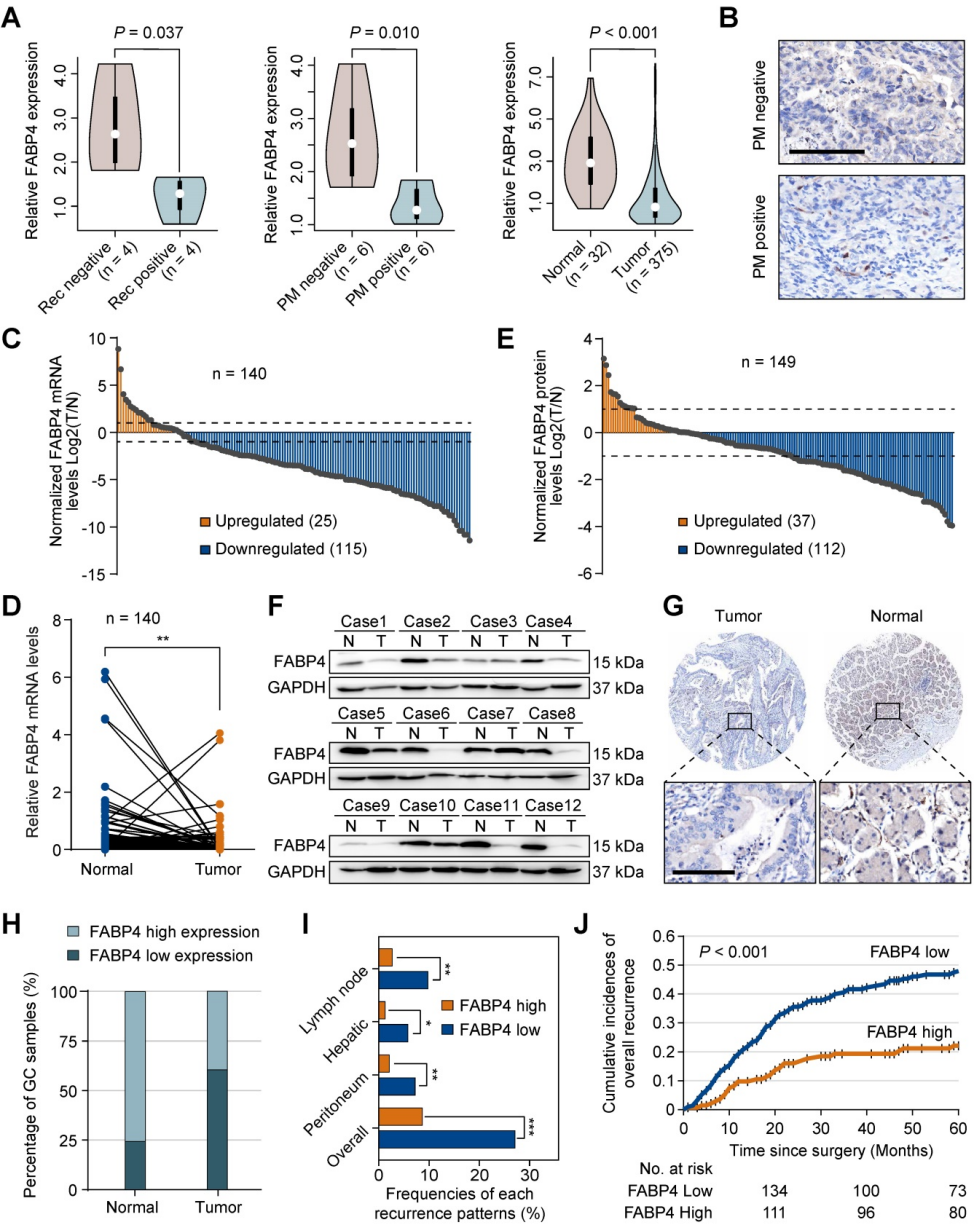
**Aberrant expression of FABP4 in human GC and its clinical significance. (A)** Violin plots showing FABP4 expression in the 3 datasets. **(B)** Representative images of FABP4 staining by IHC in primary tumours from patients with peritoneal metastasis and with nonperitoneal metastasis. Scale bars, 100 μm. **(C-D)** Real-time PCR analysis of FABP4 expression in GC and adjacent normal tissues (n = 140). **(E)** The tumour-to-normal T/N ratios of FABP4 expression at the protein level in 149 pairs of GC and adjacent normal tissues. **(F)** Representative images of FABP4 protein levels in 149 paired GC tissues. **(G)** FABP4 expression detected by IHC staining in 352 specimens from the internal cohort. Scale bars, 100 μm. **(H)** Distribution of FABP4 expression in normal gastric tissues and matched GC tissues. **(I)** Association between FABP4 expression and the frequency of all recurrence patterns. **(J)** Cumulative incidence of overall recurrence was significantly greater in GC patients with low FABP4 expression than that in patients with high FABP4 expression. The data are presented as the mean ± SD (*P < 0.05; **P < 0.01). PM, peritoneal metastasis. Rec, recurrence.

To explore specific biomarkers for the prediction of GC metastasis, we performed RNA sequencing (RNA-seq) in the samples of 20 patients with and without the presence of PM or recurrence. A subset of differentially expressed genes was identified based on the fold-change ≥ 2 and *P*-value < 0.05. Subsequently, we used the TCGA-STAD dataset to narrow down the list of the candidate genes and identified 5 overlapping biomarkers (FABP4, FHL1, LARGE2, ASCL2, and VIL1). FABP4 was the most extensively investigated gene in cancer out of this list. However, the roles of FABP4 in GC remain unknown. We detected the expression of FABP family members in RNA-seq (**Figure S1B**), which showed that only FABP4 reached a statistical significance. Therefore, we selected FABP4 for subsequent experiments (**Figure 1A**). The data of immunohistochemistry (IHC) staining (**Figure 1B**) confirmed that FABP4 was downregulated in GC patients with PM. In addition, we took 3 cases of GC patients with high FABP4 as positive controls (**Figure S1C**).

FABP4 was expressed in normal gastric tissues; in GC both the mRNA (**Figure 1C**-**D**) and protein levels of FABP4 (**Figure 1E**-**F**) were substantially decreased. Additionally, we systematically evaluated the prognostic significance of FABP4 levels in tissue specimens in an internal cohort of GC patients (n = 352). A strong positive FABP4 signal was detected in normal tissues, and a clear decrease in FABP4 signal was observed in tumour tissues (**Figure 1G-H**). Interestingly, clinicopathological analysis demonstrated that a decrease in FABP4 expression was correlated with aggressive clinical and pathological characteristics, including increased tumour size, decreased differentiation status and advanced TNM stage (**Table S2**). Patients with low FABP4 expression had worse outcomes than patients with high FABP4 expression (**Figure S2A**). Multivariate Cox regression analysis revealed that FABP4 expression was an independent prognostic factor for overall survival of patients with GC (**Figure S2B**). Furthermore, we analysed the associations of the recurrence patterns with FABP4 expression in GC patients who underwent curative gastrectomy. The results confirmed that low FABP4 expression was significantly associated with lymph node, hepatic, peritoneal and overall recurrence patterns (**Figure 1I**). Cumulative incidence of overall recurrence in the low FABP4 expression group was greater than that in the high FABP4 expression group (**Figure 1J**). To validate the efficacy of FABP4 as a marker in different populations, we assessed FABP4 levels in an external validation cohort (n = 123) and obtained similar results (**Table S2** and **Figure S2C-D**). These data indicated that FABP4 expression was associated with GC recurrence and patient outcomes.

### FABP4 attenuates GC metastasis *in vitro and in vivo*

The effects of FABP4 on malignant properties were evaluated in human GC cell lines (AGS, BGC-823 and MGC-803) with stable overexpression or knockdown of FABP4. At the same time, we took FABP5 as a negative control, and observed no significant change in the expression of FABP5 (**Figure S3A-C**). While ectopic expression of FABP4 significantly inhibited the migration and invasion of GC cells (**Figure S3D**) and increased the adhesion of the cells *in vitro* (**Figure S3E**). Conversely, inhibition of FABP4 expression in MGC-803 and AGS cells elicited opposite effects (**Figure S3F-G**). However, overexpression or knockdown of FABP4 did not influence the proliferation or apoptosis of GC cells *in vitro (***Figure S3H-K**).

**Figure 2:**
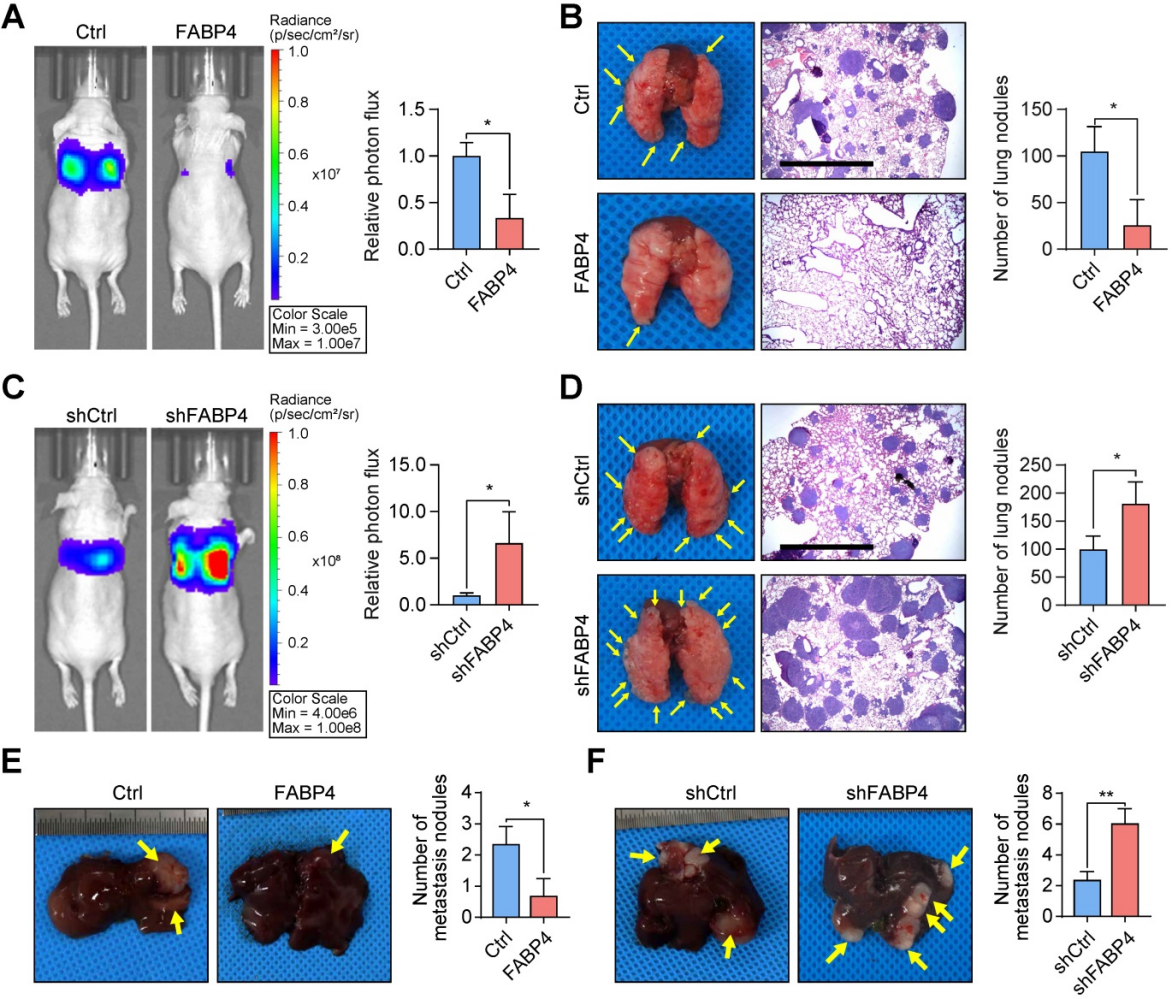
** FABP4 considerably suppresses the metastatic ability of GC cells. (A-D)** An *in vivo* lung metastatic model was generated using stably transfected BGC-823 cells (n = 3 for each mouse group). Representative images of mice and the results of quantification of the lesions are presented. Ectopic expression of FABP4 reduced the number of metastatic nodules, and knockdown of FABP4 increased the number of metastatic nodules. Scale bars, 1 mm. **(E-F)** Stably transfected MGC-803 cells were injected into the spleen to generated an *in vivo* model of liver metastasis (n = 3 for each mouse group). Representative images of mice and metastatic nodules are shown. The data are presented as the mean ± SD (*P < 0.05; **P < 0.01).

To further test the *in vivo effects* of FABP4 on metastatic potential, a lung metastatic model was generated. It was found that upregulation of FABP4 suppressed GC cell metastasis (mice with FABP4-overexpressing tumours showed significantly lower number of metastatic colonies in the lungs than mice with tumours that did not overexpress) FABP4 (**Figure 2A-B**), while downregulation of FABP4 expression increased metastasis (**Figure 2C-D**). In addition, we injected GC cells into the spleen of nude mice to induce the formation of metastases in the liver. Examination of dissected livers revealed a lower number of tumour foci in the FABP4 overexpression group than that in the control group (**Figure 2E**), and FABP4 knockdown resulted in an opposite trend (**Figure 2F**). In a subcutaneously implanted GC tumour model, consistent with the *in vitro* results, FABP4 had no effect on the growth of the xenografts (**Figure S4A-F**). Overall, these results suggest that FABP4 functions as a strong suppressor of GC metastasis and does not promote tumour growth.

### CADM3 is a critical function-related target of FABP4

**Figure 3:**
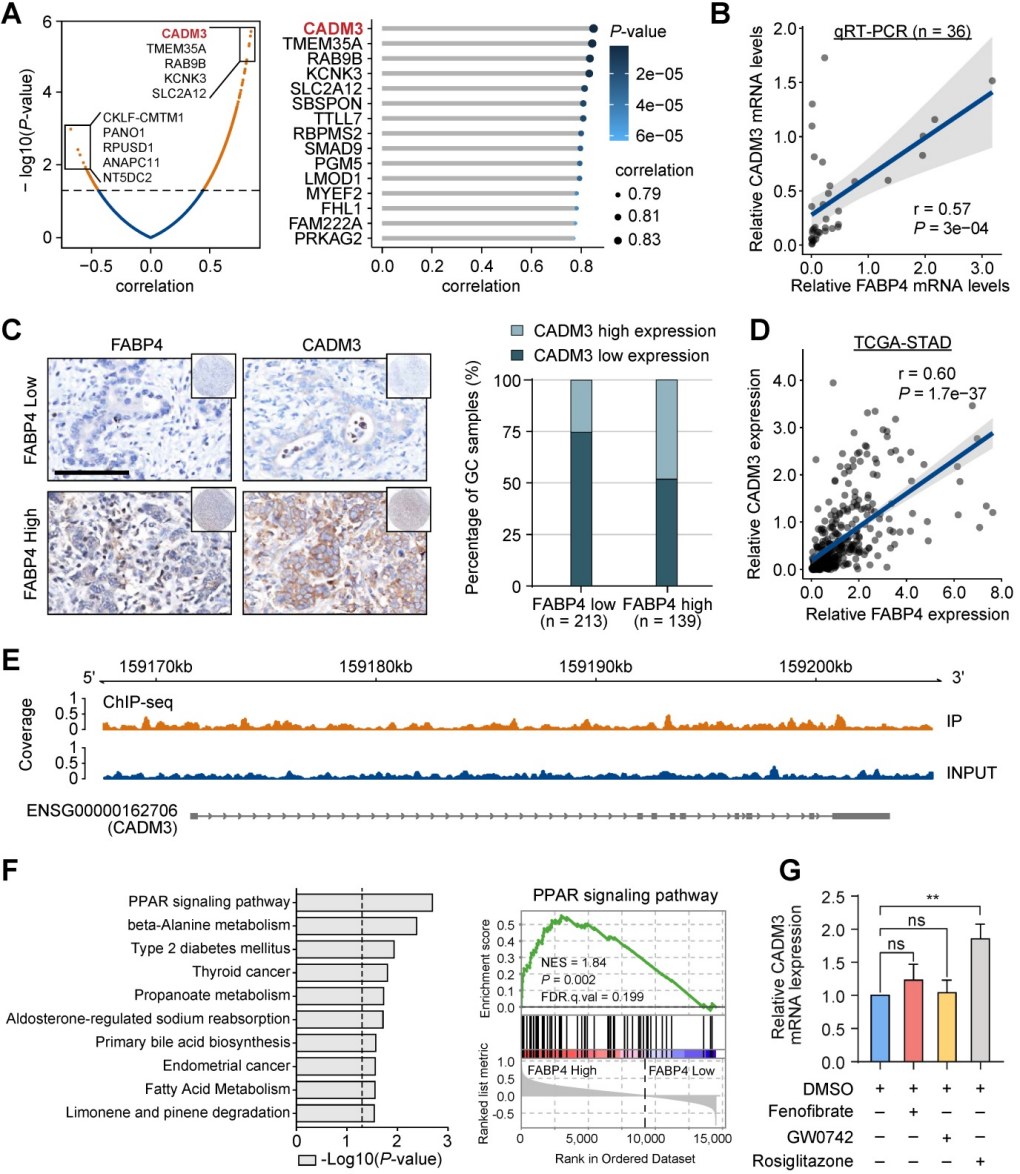
** Identification of CADM3 as a function-related target of FABP4. (A)** Volcano plot (left panel) and lollipop chart (right panel) showing the correlations between FABP4 the differentially expressed genes identified by RNA-seq. **(B)** The results of real-time PCR verifying that the mRNA levels of FABP4 and CADM3 were significantly positively correlated in 36 GC samples. **(C)** Representative images of FABP4 and CADM3 staining in tissue microarrays are presented (left panel), and CADM3 expression in various FABP4 groups of the internal cohort (right panel) was calculated. **(D)** Scatter plots showing the correlations between FABP4 and CADM3 expression levels in the TCGA-STAD dataset. **(E)** Peak signals indicating that FABP4 did not directly bind to the CADM3 promoter region. **(F)** Gene set enrichment analysis of RNA-seq data and the PPAR signalling pathway is presented. **(G)** MGC-803 cells were pretreated with or without fenofibrate (50 μM), GW0742 (10 μM), and rosiglitazone (20 μM) for 24 h. The cells were used for real-time PCR to detect the effects of various PPAR subtype agonists on CADM3 transcription. The data are presented as the mean ± SD (**P < 0.01).

Given the key role of FABP4 in GC metastasis and in the prediction of clinical outcomes, we performed a bioinformatics analysis using RNA-seq to gain further insight into the underlying molecular mechanism. We compared the transcriptomes of human GC samples with high and low expression of FABP4. Interestingly, the levels of CADM3, which has been characterized as a cell adhesion gene, were mostly significantly positively correlated with the levels of FABP4 (**Figure 3A**). Both Up-regulated and down-regulated candidate genes were selected for validation in public database TCGA and GSE15459 (**Figure S5A**). Positive correlation between FABP4 and CADM3 was further confirmed by real-time PCR analysis of an independent set of 36 GC specimens (**Figure 3B**). The results of tissue microarray-based IHC analysis validated that patients in the low FABP4 expression groups in two independent cohorts had decreased CADM3 levels (**Figure 3C** and **Figure S5B**). Similar results were obtained by analysis of the TCGA-STAD and GSE15459 datasets (**Figure 3D** and **Figure S5C**).

CADM3 has been implicated in tumorigenesis in previous studies, displaying tumour-suppressive properties in glioma and several other types of cancer 18-20. However, the exact role of CADM3 in GC is unknown. Patients with low CADM3 expression had worse outcomes than patients with high CADM3 expression (**Figure S5D**). To investigate whether antitumour effects of FABP4 are mediated by CADM3, we constructed a CADM3 eukaryotic expression vector and used siRNAs targeting CADM3 (siCADM3) (**Figure S5E-F**). We also took CADM2/4 as a negative control. The results showed that CADM2/4 were not significantly changed (**Figure S5E-F**). The results indicated that the expression of CADM3 inhibited and the expression of siCADM3 increased the migration and invasion of GC cells (**Figure S5G-H**). A further rescue experiment on the role of CADM3 in FABP4-associated metastasis showed that no significant difference was found in the invasive capacity of GC cells either when FABP4 was re-introduced with CADM3 knocked down or when FABP4 was disrupted with CADM3 overexpressed, which hints that the CADM3 is the downstream of FABP4 (**Figure S5G-H**).

The relationships between FABP4 and CADM3 expression suggested a transcriptional regulation mechanism. Therefore, chromatin immunoprecipitation sequencing (ChIP-seq) was used to show the presence of a FABP4-binding site in the CADM3 promoter *in vivo*. The results indicated that the promoter region of CADM3 was not enriched in the immunoprecipitation complex generated using a specific antibody to FABP4, suggesting that FABP4 cannot directly promote the transcription of CADM3 in GC (**Figure 3E**). In addition, the results of gene set enrichment analysis (GSEA) indicated that the most significantly upregulated molecular signatures in GC samples with high FABP4 expression included peroxisome proliferator-activated receptor (PPAR) signalling pathway-related gene sets (**Figure 3F**).

PPARs are a group of ligand-dependent nuclear transcription factors, including PPAR-α, PPAR-β and PPAR-γ 21, 22. We aimed to determine whether subtypes of PPARs may influence CADM3 expression. The results of the experiments using various agonists of PPARs indicated that rosiglitazone, a PPAR-γ agonist, dramatically increased CADM3 expression at the mRNA level while other agonists had no effect on CADM3 expression (**Figure 3G**). These data indicated that CADM3 is a key molecule mediating the protective effects of FABP4 in GC. We hypothesised that inactivation of the PPAR-γ transcription factor is likely to contribute to the inhibition of CADM3 in GC with low FABP4 expression.

### FABP4 facilitates nuclear receptor PPAR-γ-regulated CADM3 expression

**Figure 4:**
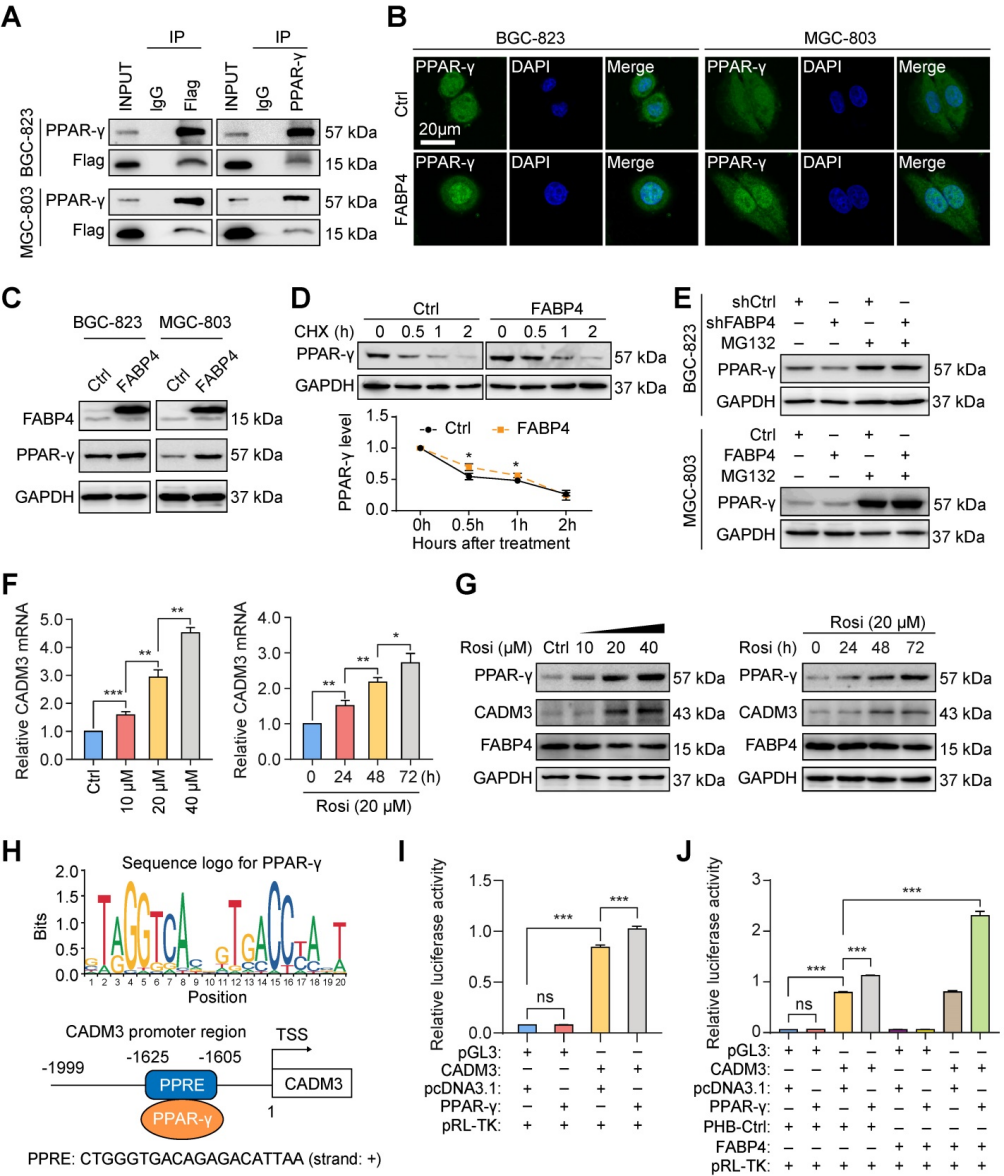
**FABP4 promotes PPAR-γ-mediated CADM3 transcription. (A)** A coimmunoprecipitation assay was performed to analyse the interaction between FABP4 and PPAR-γ in GC cells. **(B)** The results of immunofluorescence staining indicating that FABP4 facilitates the translocation of PPAR-γ from the cytoplasm to the nucleus. Scale bars, 20 μm. **(C)** The protein levels of FABP4 and PPAR-γ in GC cells measured by western blotting. **(D)** MGC-803 cells with or without altered FABP4 expression were treated with 25 μg/mL cycloheximide at indicated time points. **(E)** Stably transfected BGC-823 and MGC-803 cells were treated with or without MG132 (10 μM) for 6 h. The protein levels of PPAR-γ were analysed by western blotting. **(F-G)** MGC-803 cells were collected for real-time PCR or western blotting detection of time- and dose-dependent effects of rosiglitazone on CADM3 expression. **(H)** The PPRE region of the CADM3 promoter sequence is presented. **(I-J)** Dual-luciferase reporter assays were performed to investigate the effect of FABP4 and PPAR-γ on CADM3 transcription. The data are presented as the mean ± SD (*P < 0.05; **P < 0.01; ***P < 0.001). CHX, cycloheximide. Rosi, rosiglitazone. TSS, transcription start site. PPRE, peroxisome proliferator responsive element.

Analysis using the Search Tool for the Retrieval of Interacting Genes (STRING) database indicated that FABP4 can interact with PPAR-γ (**Figure S6A**). Previous studies have suggested that PPAR-γ often acts as a ligand-dependent transcription factor to regulate the downstream target genes 23, 24. We thus hypothesised that FABP4 is a ligand of PPAR-γ and that direct binding to PPAR-γ mediated the corresponding biological roles of FABP4. The results of the coimmunoprecipitation assays indicated that ectopically expressed 3Flag-FABP4 was immunoprecipitated with endogenous PPAR-γ in GC cells (**Figure 4A**). Immunofluorescence assays were used to analyse the effect of FABP4 expression on subcellular localization of PPAR-γ. As is shown in** Figure 4B**, ectopic expression of FABP4 induced the translocation of PPAR-γ from the cytoplasm to the nucleus.

The results of subsequent western blotting assays revealed an increase in PPAR-γ expression by FABP4 upregulation in GC cells (**Figure 4C**). Overexpression of FABP4 significantly increased the half-life of PPAR-γ (**Figure 4D**). Moreover, the results of the experiments using the peptide aldehyde proteasome inhibitor MG132 indicated that FABP4 decreased the degradation of endogenous PPAR-γ (**Figure 4E**). Moreover, rosiglitazone activated PPAR-γ in MGC-803 cells as according to the data of the western blotting assays (**Figure S6B**). Additionally, rosiglitazone time- and dose-dependently influenced the expression of CADM3 at the mRNA and protein levels (**Figure 4F-G**). However, FABP4 expression was not influenced by rosiglitazone treatment (**Figure 4G**), suggesting that PPAR-γ mediates the regulation of CADM3 expression by FABP4.

Then, we investigated whether PPAR-γ directly regulates CADM3 expression at the transcriptional level. The CADM3 promoter sequence retrieved from the UCSC Genome Browser and the JASPAR transcription factor database indicated the presence of structural characteristics of a PPRE region (**Figure 4H**). Then, we constructed the CADM3 promoter-luciferase reporter gene plasmid (**Figure S6C**). The results of the dual-luciferase reporter assays indicated that the CADM3 promoter was transactivated by PPAR-γ (**Figure 4I**), and FABP4 facilitated this effect (**Figure 4J**). These results indicated that FABP4 binds to the transcription factor PPAR-γ as a ligand and enhances its transcriptional activity, which directly promotes CADM3 expression in GC.

### Rosiglitazone is a potential antitumour agent for treatment of FABP4-deficient GC

**Figure 5:**
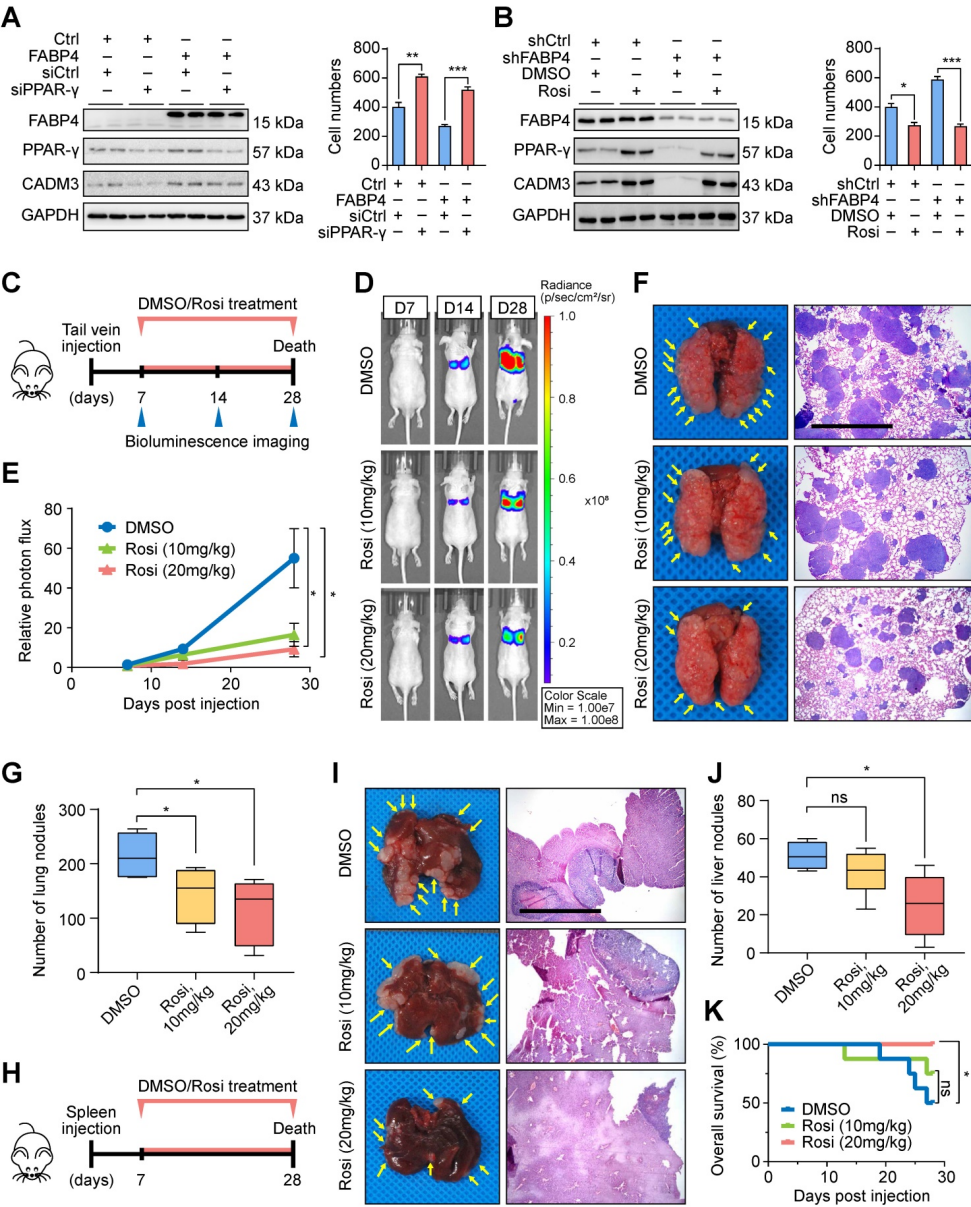
**Rosiglitazone can reverse the metastatic ability of FABP4-deficient GC cells *in vitro* and *in vivo*. (A-B)** Stably transfected MGC-803 cells were treated with siPPAR-γ and rosiglitazone (20 μM). The protein levels of FABP4, PPAR-γ and CADM3 were determined by western blotting. The migration and invasion of GC cells were determined by the Transwell assays. **(C)** Scheme of experimental design for generation of a lung metastasis model. FABP4-deficient BGC-823 cells were transplanted into the tail veins of BALB/c nude mice, followed by daily treatment with DMSO or rosiglitazone (10 mg/kg or 20 mg/kg) (5 mice per group). **(D-E)** Representative images of mice and quantified results of bioluminescence imaging are presented. **(F-G)** The lungs were collected for H&E staining, and the number of metastatic nodules was determined. Scale bars, 1 mm. **(H)** Scheme of experimental design for generation of a liver metastasis model. FABP4-deficient BGC-823 cells were transplanted into the spleens of BALB/c nude mice, followed by daily treatment with DMSO or rosiglitazone (10 mg/kg or 20 mg/kg) (8 mice per group). **(I-J)** The livers were collected for H&E staining, and the number of metastatic nodules was determined. Scale bars, 1 mm. **(K)** The survival of BALB/c nude mice was monitored. The data are presented as the mean ± SD (*P < 0.05; **P < 0.01; ***P < 0.001). Rosi, rosiglitazone.

To verify molecular mechanisms responsible for FABP4-mediated GC metastasis, we performed a series of rescue assays in MGC-803 GC cells. The results indicated that upregulation of CADM3 expression and inhibition of invasion and migration in GC cells induced by FABP4 were reduced in cells transfected with PPAR-γ siRNA (**Figure 5A** and **Figure S7A**). Importantly, downregulation of CADM3 and aggressive features of GC cells caused by FABP4 knockdown were effectively reversed by rosiglitazone (**Figure 5B** and **Figure S7B**).

Considering the important role of rosiglitazone *in vitro*, we investigated whether these findings are applicable *in vivo*. As shown in **Figure 5C**, we generated a model of BALB/c nude mice bearing the tumours derived from FABP4-deficient BGC-823 cells delivered by tail vein injection; these animals received intraperitoneal injections of rosiglitazone or vehicle DMSO. The results of bioluminescence imaging assays indicated that rosiglitazone treatment dramatically suppressed tumour metastasis compared with the DMSO treatment (**Figure 5D-E**). Examination of the lungs isolated from tumour-bearing mice on day 28 showed that rosiglitazone treatment significantly reduced the number of metastatic colonies (**Figure 5F-G**). Moreover, these cells were injected in the spleen of nude mice to generate a liver metastasis model (**Figure 5H**). The group treated with 20 mg/kg rosiglitazone manifested a decrease in the number of metastatic foci in the liver tissue sections (**Figure 5I-J**) and had a better prognosis compared with those in the control group (**Figure 5K**). Overall, these findings indicated that rosiglitazone reverses the changes in metastatic ability of FABP4-deficient GC cells *in vitro* and *in vivo*.

### Correlation between rosiglitazone treatment and the risk of recurrence in patients with GC

**Figure 6:**
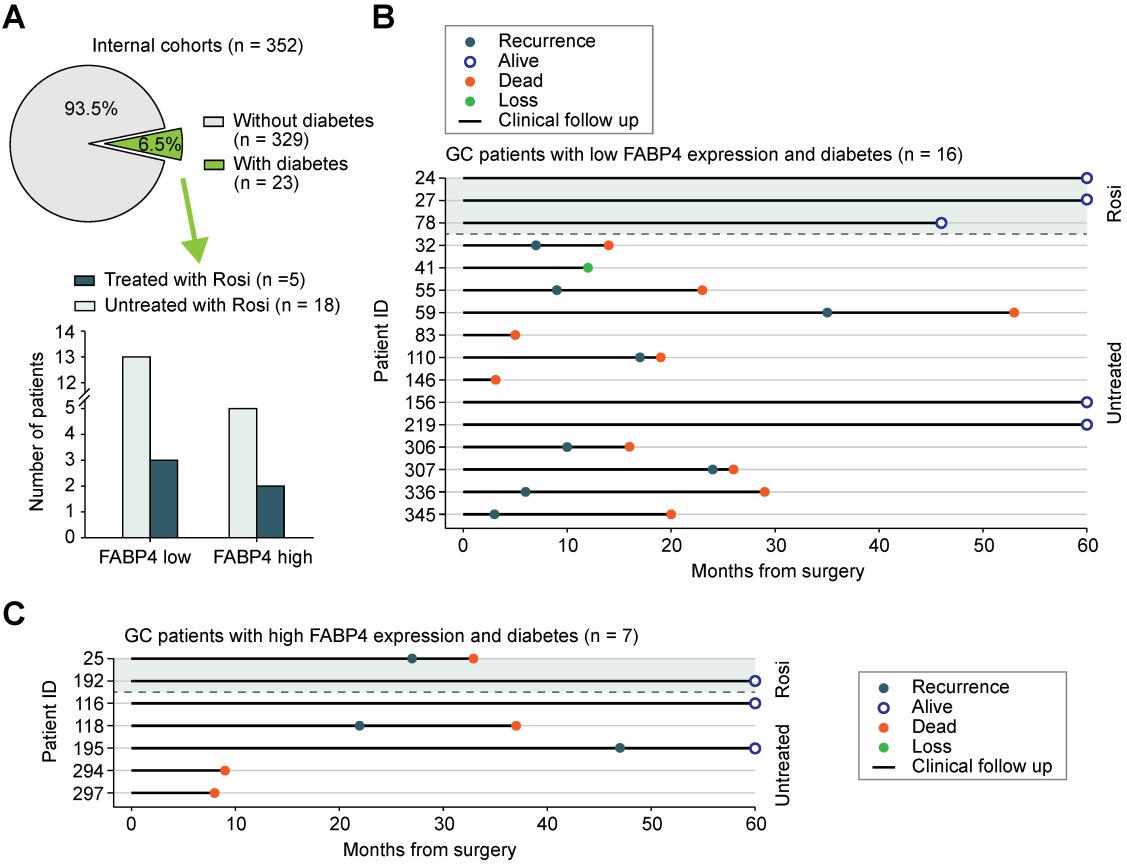
**Potential antitumour effect of rosiglitazone in patients with low FABP4 expression. (A)** The proportion and distribution of the number of patients with diabetes and patients receiving rosiglitazone treatment in GC patients of the internal cohort. **(B)** Analysis of recurrence and longitudinal survival status of FABP4-negative patients who did or did not receive rosiglitazone treatment. **(C)** Analysis of survival data from FABP4-positive patients who did or did not receive rosiglitazone treatment. Rosi, rosiglitazone.

To assess whether rosiglitazone treatment is associated with GC recurrence after curative gastrectomy, we collected additional information on the use of antidiabetic medications by patients with GC. Among 352 patients with GC in the internal cohort, 23 (6.5%) patients were diagnosed with diabetes, and 5 (21.7%) of these 23 patients received rosiglitazone (**Figure 6A**). Patients with low FABP4 expression had a lower recurrence rate or better survival after receiving rosiglitazone treatment (n = 3) than those in patients were not treated with rosiglitazone (n = 13) (**Figure 6B**). However, patients with high FABP4 expression did not benefit from rosiglitazone administration (**Figure 6C**), implying that the antitumour effect of rosiglitazone is different across individuals.

### Chromatin inaccessibility may orchestrate reduced FABP4 expression via HDAC1 overexpression

**Figure 7:**
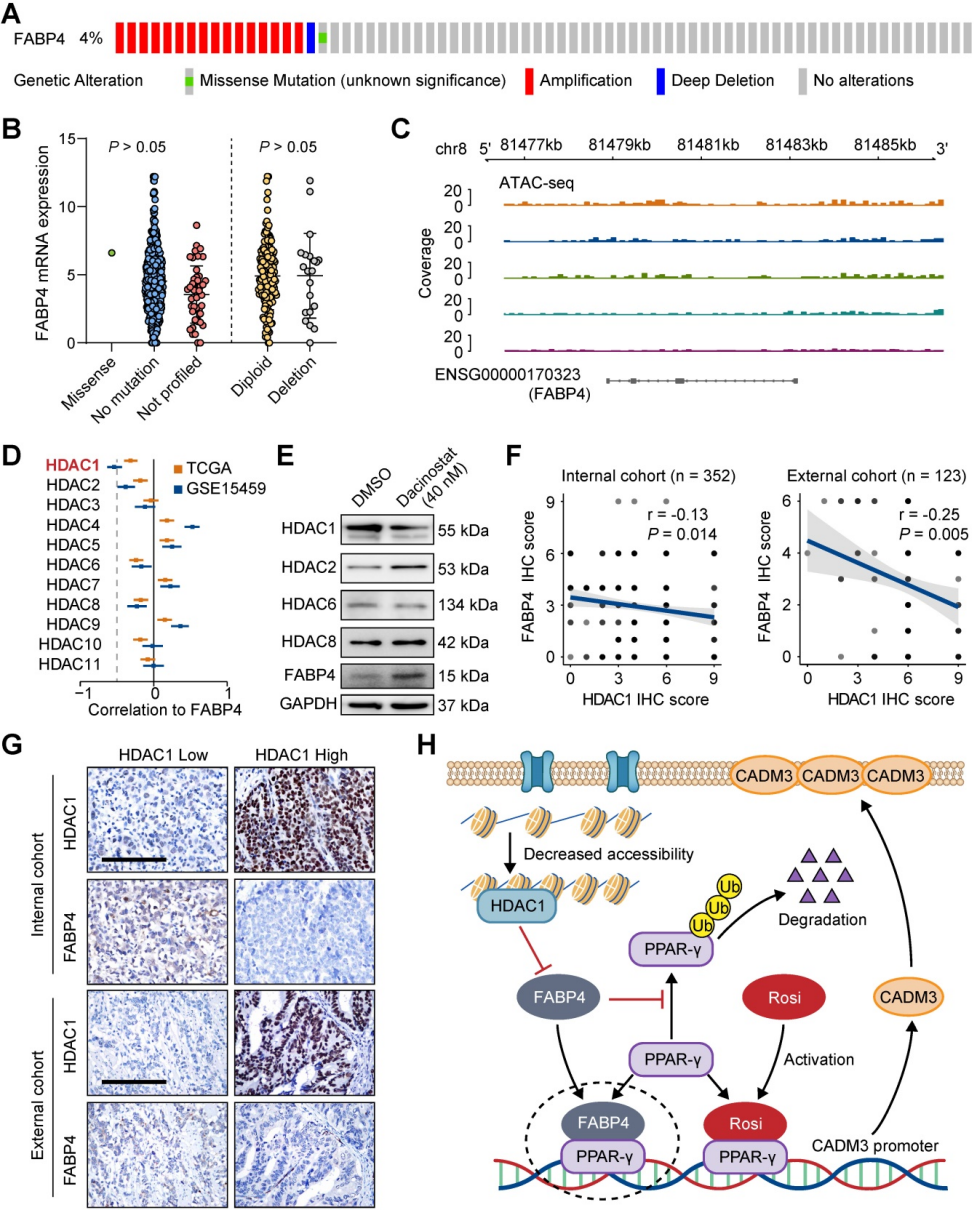
**Decreased FABP4 expression may be related to chromatin inaccessibility mediated by HDAC1. (A-B)** Summary of FABP4 mutations and CNV identified in GC patients using cBioPortal. **(C)** The peak signals indicated that FABP4 is present within a region of inaccessible chromatin. **(D)** Analyses of the correlation between FABP4 expression and HDAC gene expression in the TCGA-STAD and GSE15459 datasets. **(E)** BGC-823 cells were treated with or without dacinostat (40 nM) for 24 h. **(F-G)** Representative images of FABP4 and PPAR-γ staining by IHC are presented. The correlation between FABP4 and PPAR-γ was calculated. Scale bars, 100 μm. **(H)** Schematic illustration of the role of FABP4 in the regulation of GC metastasis.

Since downregulated FABP4 expression was detected in GC tissues compared with that in normal tissues, we were interested in what causes this dysregulation of FABP4. We used cBioPortal to analyse the frequency and type of FABP4 alterations in a TCGA dataset. Further analysis showed that copy number variation (CNV) or mutations were not the main cause of altered FABP4 expression (**Figure 7A-B** and **Figure S8A**). In addition, FABP4 expression was not associated with methylation of its promoter (**Figure S8B**), and hypermethylated CpG sites were not identified by MethPrimer analysis.

An assay for transposase-accessible chromatin by high-throughput sequencing (ATAC-seq) analysis unexpectedly revealed that FABP4 had low chromatin accessibility in the GC tissues (**Figure 7C**), implying that low expression of FABP4 in GC may be caused by decreased chromatin accessibility and not by CNV, mutations or DNA methylation (**Figure S8C**). Considering that the activity of histone deacetylases (HDACs) is closely linked to chromatin accessibility and aberrant expression of the genes 25, 26, we analysed correlations between HDACs and FABP4 in both TCGA and GSE15459 datasets. Only the expression of HDAC1 was notably correlated with the expression of FABP4 (**Figure 7D**). The HDAC inhibitor dacinostat (LAQ824) was used to detect alterations in of FABP4 expression. The results showed an increase in FABP4 expression after HDAC1 inhibition, and the expression levels of additional HDACs showed no significant change (**Figure 7E**). Matched scatterplots showed that the correlations between HDAC1 and FABP4 and HDAC1 and CADM3 expression (**Figure S8D**) in GC tissues were proportional. Negative correlations between HDAC1 and FABP4 and between HDAC1 and CADM3 were confirmed in two independent cohorts of 475 GC specimens (**Figure 7F-G** and **Figure S8E**). Together, these results demonstrated that downregulated expression of FABP4 in GC was caused by decreased accessibility and was correlated with HDAC1.

## Discussion

GC patients with metastasis or recurrence always suffer low quality of life due to the lack of clinically effective treatment approaches 27-30. Thus, in-depth studies on GC metastasis and recurrence at the molecular level are urgently needed to identify novel prevention and intervention strategies based on* in vivo* and *in vitro* analyses. The present study used RNA-seq to identify FABP4 as a specific gene associated with GC metastasis and peritoneal recurrence and further confirmed these findings by IHC of GC patient samples. Recent studies reported that the roles of FABP4 vary in different tumors. For example, FABP4 promotes tumour cell progression via the IL-6/STAT3/ALDH1 axis in obesity-associated breast cancer. Inhibition of FABP4 effectively prevented early metastasis of ovarian cancer. In contrast, FABP4 exhibited an antitumour effect on hepatocellular carcinoma cells. Inconsistent results of the same index in different cancers have also been reported in the literature, such as the Sox2 31-36. The effect of the same genes on tumorigenesis is highly context-dependent, probably because the same indicators play different roles in different organs and environments due to the involvement of different synergistic molecules. It should be noted that tumor development is a combination of multiple factors and genes. The reason why the high expression of FABP4 did not work in 20-30% GC patients was that its anticancer effect did not dominate and failed to suppress the pro-carcinogenic effect of other mutated genes. To date, only a few studies investigated the FABP4 protein in digestive tract tumours, and the role of FABP4 in GC has rarely been explored. To the best of our knowledge, this is the first study to intensively describe the expression, role, and mechanism of action of FABP4 in GC. The results indicated that FABP4 was downregulated in human GC tissues and was associated with GC recurrence and patient outcomes. Additionally, FABP4 significantly suppressed the invasion and migration but had no effect on the growth of tumour cells. These findings suggest that FABP4 is a potential target for GC prevention and therapy.

Rigorous bioinformatic analysis was performed to clarify the mechanisms by which FABP4 inhibits GC metastasis. We found that FABP4 was positively correlated with CADM3, and the results were confirmed using real-time PCR and public datasets. CADM3, also known as NECL-1, belongs to the nectin family, is predominantly expressed on the cell membrane and mediates calcium-independent cell-cell adhesion 18-20. Similar to FABP4, CADM3 can inhibit the invasion and metastasis of GC cells, suggesting that CADM3 may play a major role in the protective effects of FABP4. Interestingly, the results of the present study indicated that FABP4 was unable to directly bind to the CADM3 promoter as a transcription factor, and the PPAR-γ agonist rosiglitazone significantly promoted the expression of CADM3. We further demonstrated that FABP4 binds to PPAR-γ, inhibits its ubiquitin-mediated degradation and promotes its translocation to the nucleus. PPAR-γ is a well-known ligand-dependent nuclear transcription factor 23, 24. PPAR-γ binding to specific ligands changes the spatial conformation of the dimer, which then regulates the expression of the target genes via the PPRE sequence 37, 38. We found that the CADM3 promoter contains the structural characteristics of a PPRE sequence and demonstrated that CADM3 is a direct transcriptional target of PPAR-γ and that FABP4 can upregulate the transcriptional activation of CADM3 by PPAR-γ. Therefore, we suggest that FABP4 binds to PPAR-γ as a coactivator, promotes the transcriptional activation of CADM3 by PPAR-γ and thus inhibits GC metastasis.

Rosiglitazone, an insulin sensitizer, has some side effects, including cardiovascular risk, which limits clinical use of the drug 39. Recent studies have demonstrated strong therapeutic potential of rosiglitazone for antitumour therapy 40, and the use of low doses rosiglitazone can avoid known side effects of the drug 41. Therefore, standardisation of the use of rosiglitazone is extremely important. Some studies accidentally found that rosiglitazone has beneficial effect in GC treatment 42, 43; however, other studies have shown that antitumour effect of the drug is uncertain 44, 45. Therefore, patients that benefit the most from this antidiabetic drug should be adequately identified. However, suitable markers are absent for patients stratification according to response to this type of therapy. Interestingly, we found that rosiglitazone can decrease the invasion and metastatic ability of FABP4-deficient GC cells in a dose- and time-dependent manner by upregulating CADM3 expression both *in vitro* and *in vivo*. We further evaluated the effect of rosiglitazone treatment on recurrence in patients with GC, and the results showed that patients with low FABP4 expression treated with rosiglitazone had a better prognosis. However, due to limited number of patients who received rosiglitazone in the present study, the clinical value of rosiglitazone requires additional evaluation in a randomised controlled trial. The present study confirmed the antitumour effect of rosiglitazone at many levels, and this drug mainly benefits the patients with FABP4-deficient GC, which provides a reference for future investigations of rosiglitazone for antitumour therapy.

Strong antitumour effects of FABP4 suggested subsequent exploration of the regulatory mechanism of FABP4 silencing in GC using the TCGA dataset to analyse the effects of CNV, mutations, and DNA methylation on FABP4 expression; however, the results indicated a lack of significant correlations. Nevertheless, the results of ATAC-seq analysis showed that chromatin inaccessibility of FABP4 may be an important factor responsible for low expression of FABP4 in GC. Previous studies demonstrated that histone acetylation and deacetylation are vital epigenetic processes that regulate gene expression due to remodelling of chromatin accessibility 46, 47. There are 11 subtypes of HDACs, and these enzymes remove acetyl groups from acetylated lysine residues on histones, resulting in a tighter structure of histone-encapsulated DNA, which affects gene expression 26, 48-50. HDAC1 was identified as the only HDAC family member that significantly negatively correlated with FABP4, suggesting that chromatin inaccessibility of FABP4 may be caused by HDAC1. In addition, we identified the Sp1 transcription factor-binding sites in the FABP4 promoter region; however, whether FABP4 recruits HDAC1 by binding to Sp1 required additional studies.

## Conclusions

In summary, the present study demonstrated that FABP4 participates in GC metastasis by regulating the PPAR-γ/CADM3 signalling axis and that low expression of FABP4 in GC is closely related to HDAC1-mediated chromatin inaccessibility (**Figure 7H**). Postoperative treatment with rosiglitazone may benefit GC patients with low FABP4 expression.

## Supplementary Material

Supplementary figures and tables.

## Abbreviations

FABP4: fatty acid binding protein 4
GC: gastric cancer
AJCC: American Joint Committee on Cancer
PM: peritoneal metastasis
RNA-seq: RNA sequencing
TCGA-STAD: The Cancer Genome Atlas Stomach Adenocarcinoma
qRT-PCR: quantitative reverse transcription PCR
GEO: Gene Expression Omnibus
IHC: immunohistochemistry
TMA: tissue microarray
HR: hazard ratios
ChIP-seq: chromatin immunoprecipitation sequencing
GSEA: gene set enrichment analysis
PPAR: peroxisome proliferator-activated receptor
STRING: Search Tool for the Retrieval of Interacting Genes
CNV: copy number variation
ATAC-seq: assay for transposase-accessible chromatin with high-throughput sequencing
HDACs: histone deacetylases
CHX: cycloheximide
Rosi: rosiglitazone
TSS: transcription start site
PPRE: peroxisome proliferator responsive element
