# Supplementary material for: The peroxisome proliferator-activated receptor agonist rosiglitazone specifically represses tumour metastatic potential in chromatin inaccessibility-mediated FABP4-deficient gastric cancer
Source: Theranostics. 2022 Jan 24;12(4):1904–20. doi: 10.7150/thno.66814 (PMC8825587; doi:10.7150/thno.66814)
Supplement: Supplementary file 1 — Supplementary figures and tables. [file thnov12p1904s1.pdf]

Figure S1

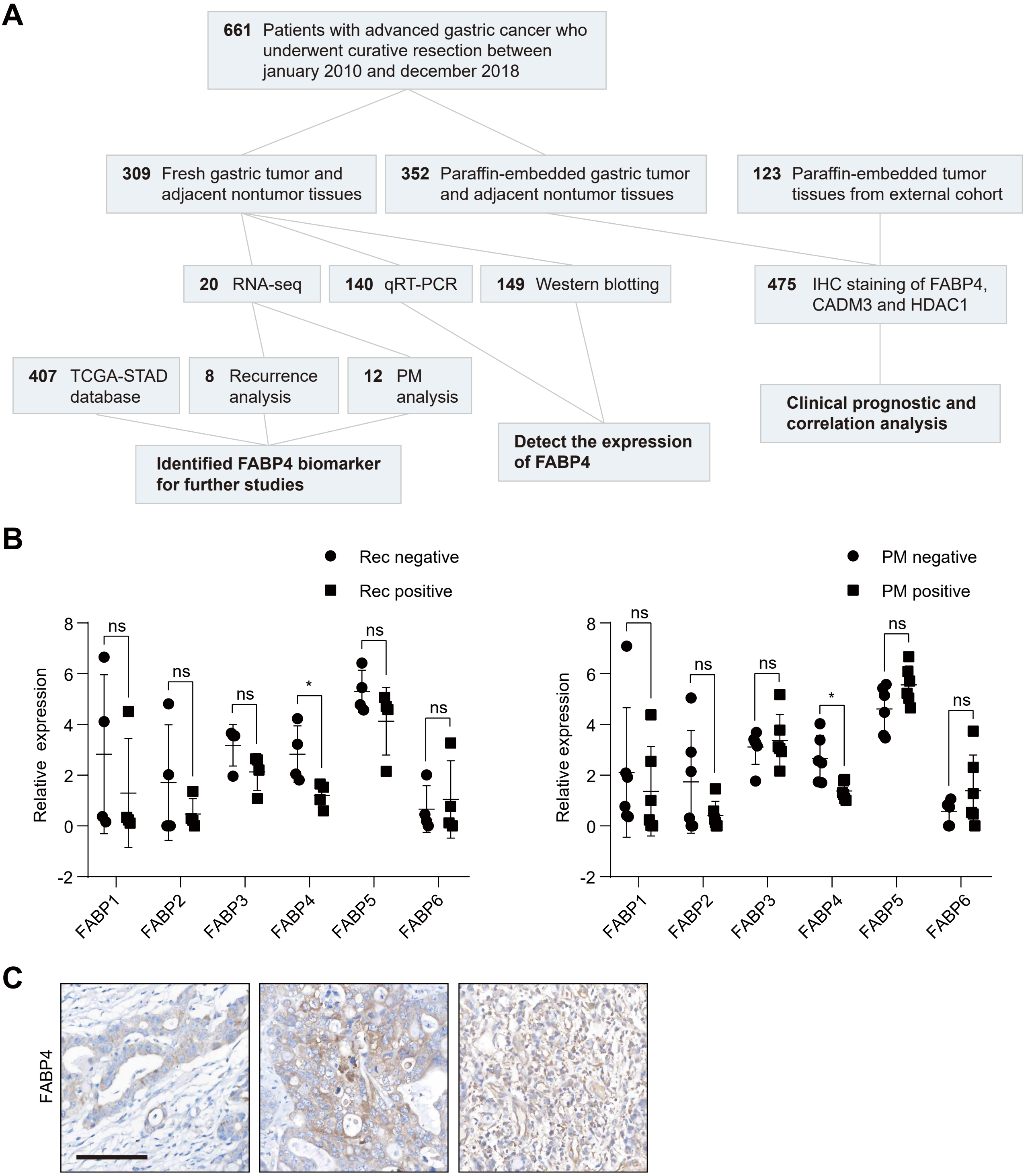
A
661 Patients with advanced gastric cancer who underwent curative resection between january 2010 and december 2018
309 Fresh gastric tumor and adjacent nontumor tissues
352 Paraffin-embedded gastric tumor and adjacent nontumor tissues
123 Paraffin-embedded tumor tissues from external cohort
20 RNA-seq
140 qRT-PCR
149 Western blotting
475 IHC staining of FABP4, CADM3 and HDAC1
407 TCGA-STAD database
8 Recurrence analysis
12 PM analysis
Clinical prognostic and correlation analysis
Detect the expression of FABP4
Identified FABP4 biomarker for further studies
B
Rec negative
Rec positive
PM negative
PM positive
Relative expression
ns
*
FABP1
FABP2
FABP3
FABP4
FABP5
FABP6
C
FABP4

Figure S2

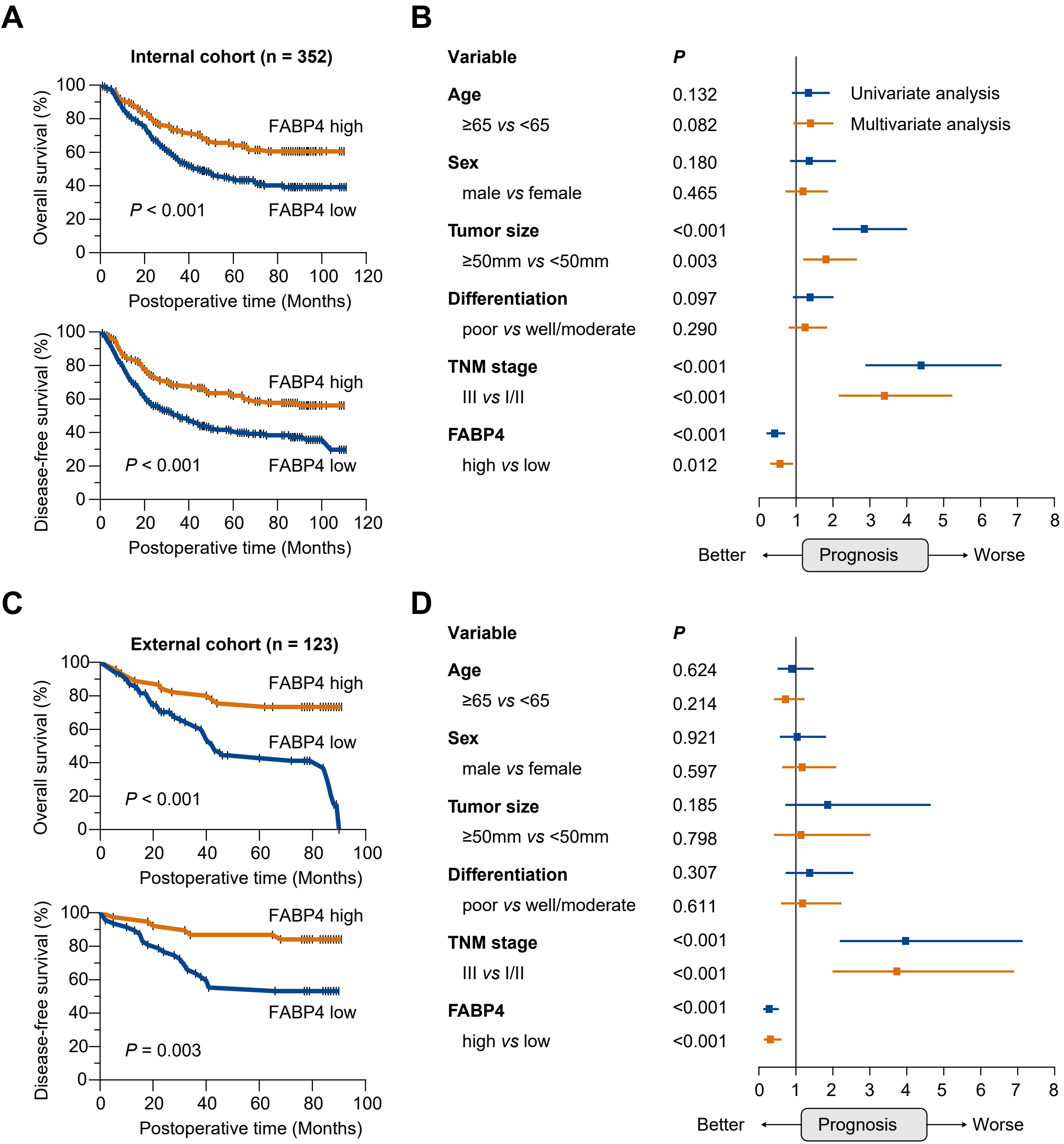
A
Internal cohort (n = 352)
Overall survival (%)
FABP4 high
FABP4 low
P < 0.001
Postoperative time (Months)
Disease-free survival (%)
FABP4 high
FABP4 low
P < 0.001
Postoperative time (Months)
B
Variable
P
Univariate analysis
Multivariate analysis
Age 0.132
≥65 vs <65 0.082
Sex 0.180
male vs female 0.465
Tumor size <0.001
≥50mm vs <50mm 0.003
Differentiation 0.097
poor vs well/moderate 0.290
TNM stage <0.001
III vs I/II <0.001
FABP4 <0.001
high vs low 0.012
Better
Prognosis
Worse
C
External cohort (n = 123)
Overall survival (%)
FABP4 high
FABP4 low
P < 0.001
Postoperative time (Months)
Disease-free survival (%)
FABP4 high
FABP4 low
P = 0.003
Postoperative time (Months)
D
Variable
P
Age 0.624
≥65 vs <65 0.214
Sex 0.921
male vs female 0.597
Tumor size 0.185
≥50mm vs <50mm 0.798
Differentiation 0.307
poor vs well/moderate 0.611
TNM stage <0.001
III vs I/II <0.001
FABP4 <0.001
high vs low <0.001
Better
Prognosis
Worse

Figure S3

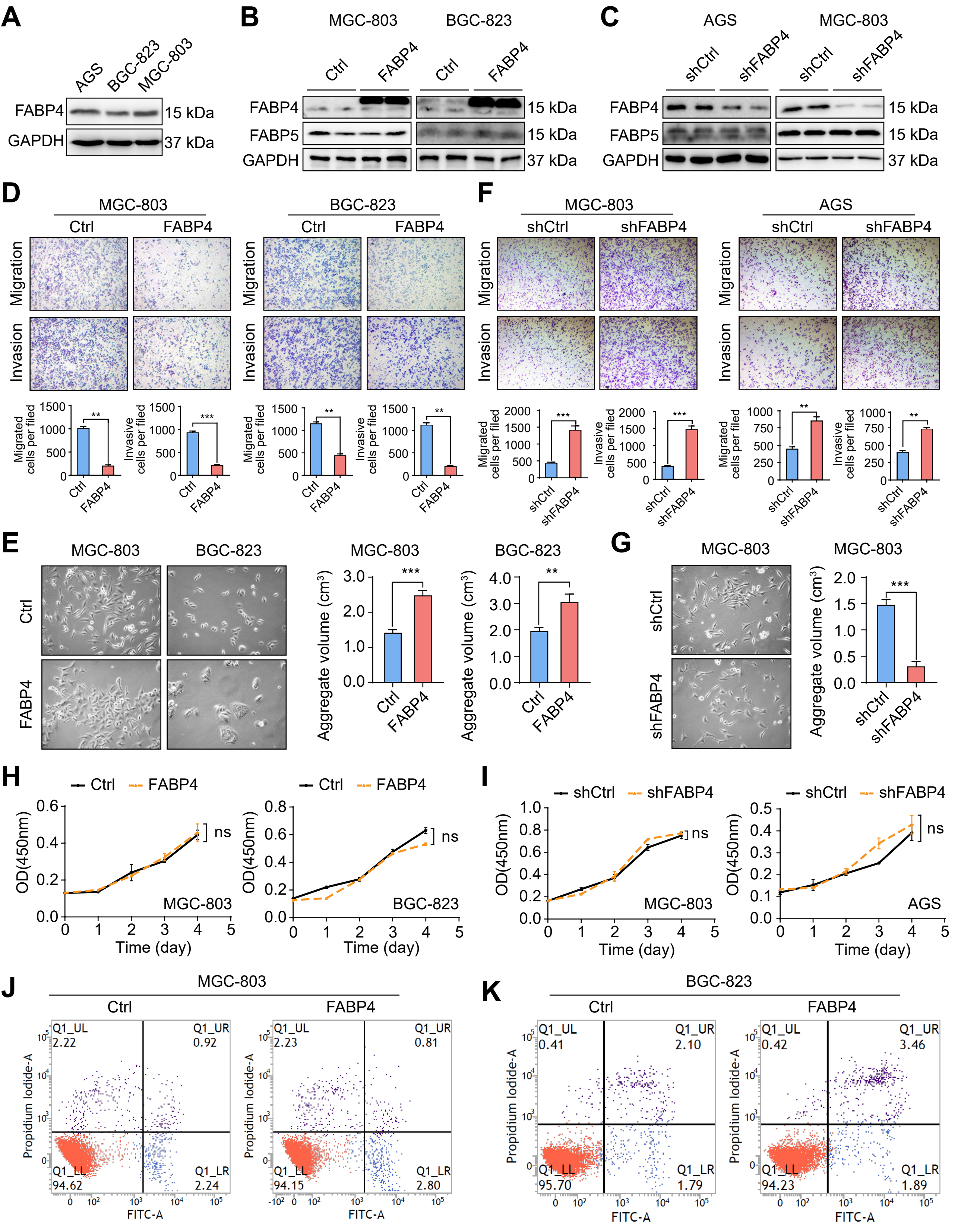
A
AGS
BGC-823
MGC-803
FABP4
15 kDa
GAPDH
37 kDa
B
MGC-803
BGC-823
Ctrl
FABP4
FABP5
C
AGS
shCtrl
shFABP4
D
Migration
Invasion
Migrated cells per filed
Invasive cells per filed
E
Aggregate volume (cm³)
F
G
H
OD(450nm)
Time (day)
ns
I
J
K
Q1_UL
Q1_UR
Q1_LL
Q1_LR
Propidium Iodide-A
FITC-A
2.22
0.92
94.62
2.24
2.23
0.81
94.15
2.80
0.41
2.10
95.70
1.79
0.42
3.46
94.23
1.89

Figure S4

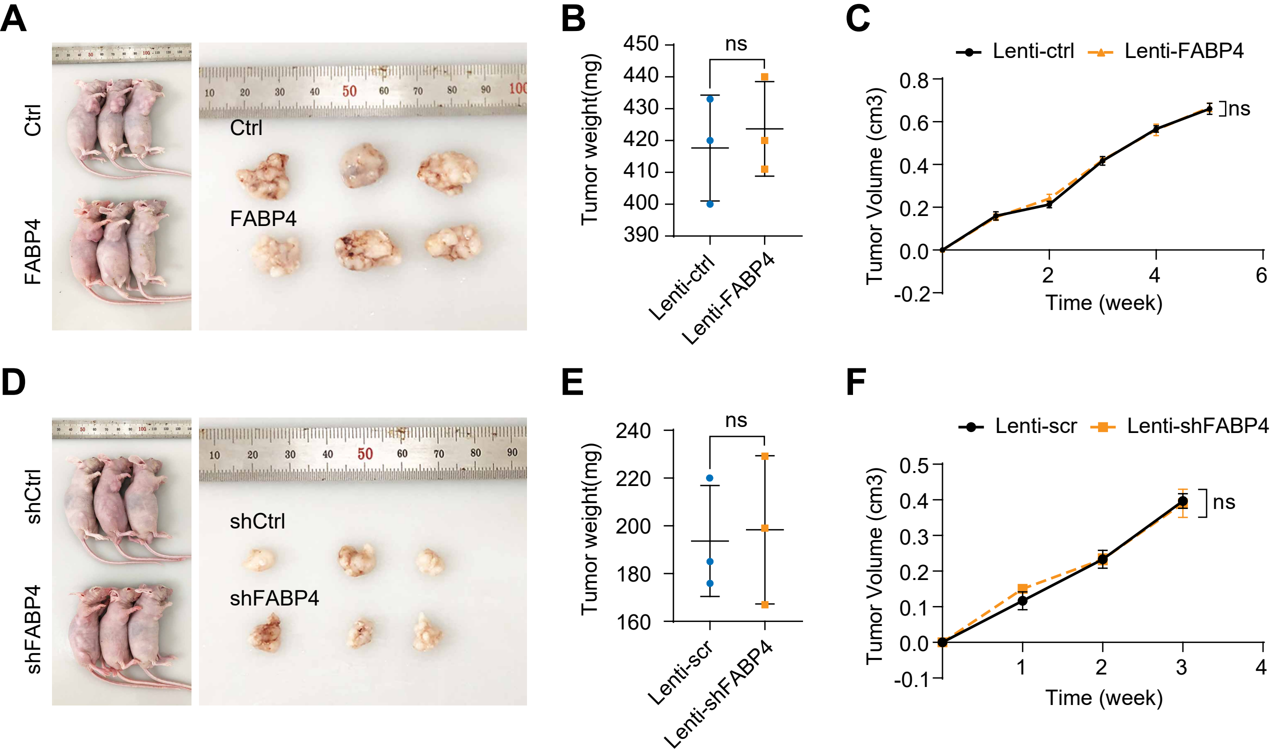
A
Ctrl
FABP4
Ctrl
FABP4
B
ns
Tumor weight(mg)
450
440
430
420
410
400
390
Lenti-ctrl
Lenti-FABP4
C
Lenti-ctrl
Lenti-FABP4
ns
Tumor Volume (cm3)
0.8
0.6
0.4
0.2
0.0
-0.2
2
4
6
Time (week)
D
shCtrl
shFABP4
shCtrl
shFABP4
E
ns
Tumor weight(mg)
240
220
200
180
160
Lenti-scr
Lenti-shFABP4
F
Lenti-scr
Lenti-shFABP4
ns
Tumor Volume (cm3)
0.5
0.4
0.3
0.2
0.1
0.0
-0.1
1
2
3
4
Time (week)

Figure S5

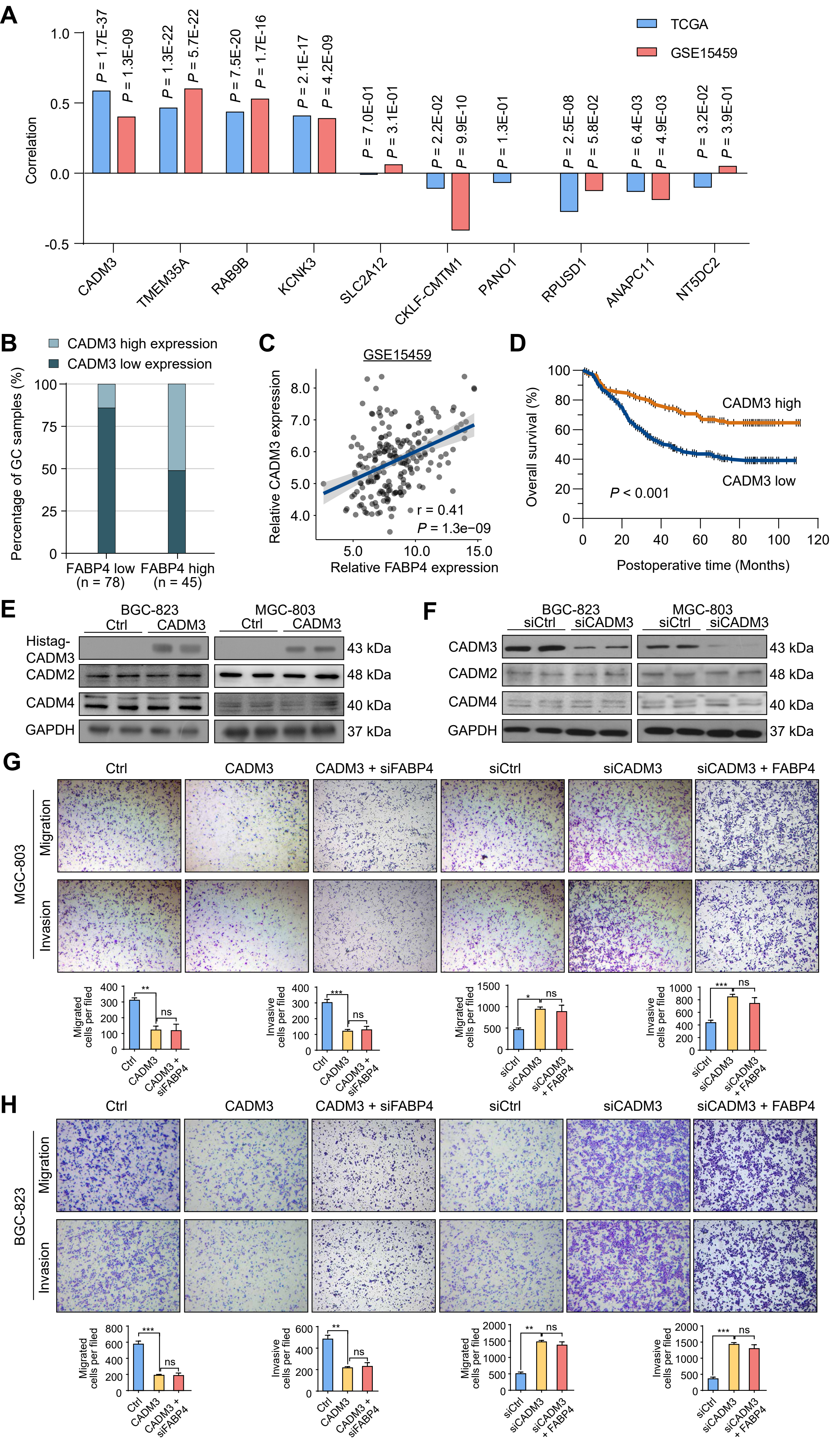
A
TCGA
GSE15459
Correlation
CADM3
TMEM35A
RAB9B
KCNK3
SLC2A12
CKLF-CMTM1
PANO1
RPUSD1
ANAPC11
NT5DC2
B
CADM3 high expression
CADM3 low expression
Percentage of GC samples (%)
FABP4 low (n = 78)
FABP4 high (n = 45)
C
GSE15459
Relative CADM3 expression
Relative FABP4 expression
r = 0.41
P = 1.3e−09
D
Overall survival (%)
CADM3 high
CADM3 low
P < 0.001
Postoperative time (Months)
E
BGC-823
MGC-803
Ctrl
CADM3
Histag-CADM3
CADM2
CADM4
GAPDH
43 kDa
48 kDa
40 kDa
37 kDa
F
siCtrl
siCADM3
G
CADM3 + siFABP4
siCADM3 + FABP4
Migration
Invasion
Migrated cells per filed
Invasive cells per filed
ns
H

Figure S6

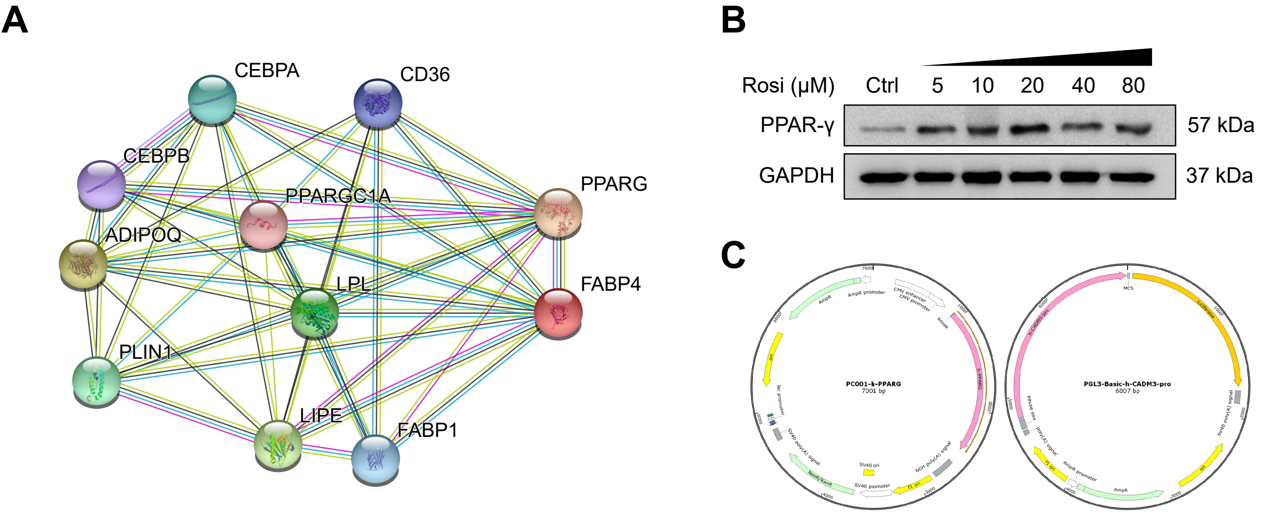
A
CEBPA
CD36
CEBPB
PPARGC1A
PPARG
ADIPOQ
LPL
FABP4
PLIN1
LIPE
FABP1
B
Rosi (μM)
Ctrl
5
10
20
40
80
PPAR-γ
57 kDa
GAPDH
37 kDa
C
PC001-h-PPARG
7001 bp
AmpR
AmpR promoter
CMV enhancer
CMV promoter
kozak
h-PPARG
bGH poly(A) signal
f1 ori
SV40 promoter
SV40 ori
NeoR/KanR
SV40 poly(A) signal
lac promoter
ori
PGL3-Basic-h-CADM3-pro
6807 bp
MCS
luciferase
SV40 poly(A) signal
ori
AmpR
AmpR promoter
f1 ori
poly(A) signal
pause site
h-CADM3-pro

Figure S7

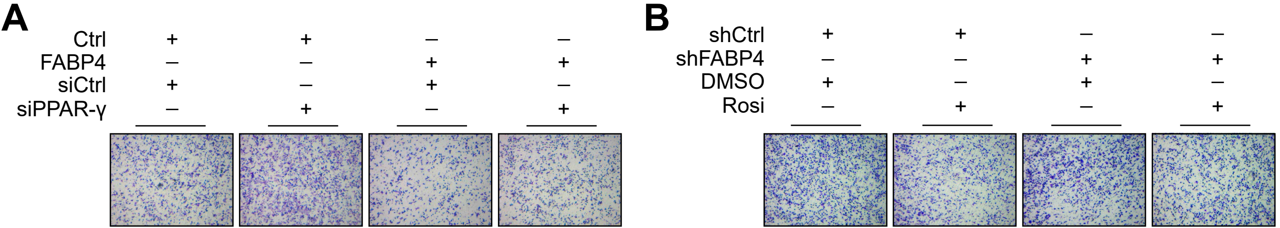

Figure S8

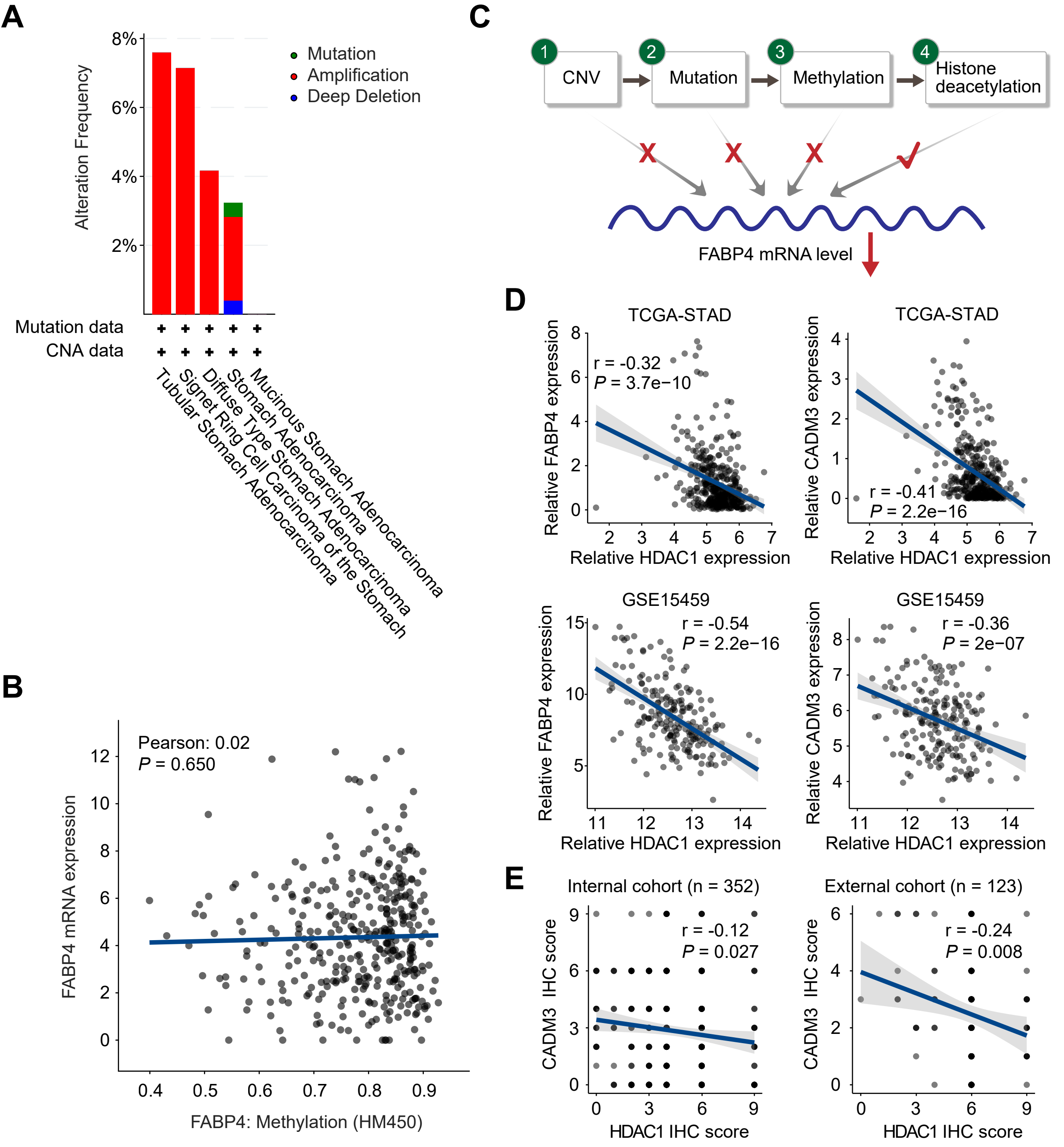

A
Alteration Frequency
8%
6%
4%
2%
Mutation
Amplification
Deep Deletion
Mutation data
CNA data
Tubular Stomach Adenocarcinoma
Signet Ring Cell Carcinoma of the Stomach
Diffuse Type Stomach Adenocarcinoma
Stomach Adenocarcinoma
Mucinous Stomach Adenocarcinoma
B
Pearson: 0.02
P = 0.650
FABP4 mRNA expression
FABP4: Methylation (HM450)
C
1 CNV
2 Mutation
3 Methylation
4 Histone deacetylation
FABP4 mRNA level
D
TCGA-STAD
r = -0.32
P = 3.7e−10
Relative FABP4 expression
Relative HDAC1 expression
TCGA-STAD
r = -0.41
P = 2.2e−16
Relative CADM3 expression
Relative HDAC1 expression
GSE15459
r = -0.54
P = 2.2e−16
Relative FABP4 expression
Relative HDAC1 expression
GSE15459
r = -0.36
P = 2e−07
Relative CADM3 expression
Relative HDAC1 expression
E
Internal cohort (n = 352)
r = -0.12
P = 0.027
CADM3 IHC score
HDAC1 IHC score
External cohort (n = 123)
r = -0.24
P = 0.008
CADM3 IHC score
HDAC1 IHC score

Table S1. Primers used for qRT-PCR

Name	Sequence (5'-3')
FABP4-F	ACTGGGCCAGGAATTTGACG
FABP4-R	CTCGTGGAAGTGACGCCTT
CADM3-F	GCTCTGTGAACCATGAATCTCT
CADM3-R	ATCATCGCAGTTGGTGTGTATA
GAPDH-F	TGCACCACCAACTGCTTAGC
GAPDH-R	GGCATGGACTGTGGTCATGAG

Table S2. Clinical characteristics and FABP4 expression of 352 gastric cancer patients in internal cohort and 123 gastric cancer patients in external validation cohort.

**Characteristic**		**Internal cohort**					**External validation cohort**				
		**Total (case [%])**	**FABP4 low (case [%])**	**FABP4 high (case [%])**	**χ2**	***P***	**Total (case [%])**	**FABP4 low (case [%])**	**FABP4 high (case [%])**	**χ2**	***P***
Age (years)					0.205	0.651				0.168	0.682
	<65	205 [58.2]	122 [34.7]	83 [23.6]			74 [60.2]	48 [39.0]	26 [21.1]		
	≥ 65	147 [41.8]	91 [25.9]	56 [15.9]			49 [39.8]	30 [24.4]	19 [15.5]		
Gender					0.013	0.908				0.001	0.975
	Female	90 [25.6]	54 [15.3]	36 [10.2]			33 [26.8]	21 [17.1]	12 [9.8]		
	Male	262 [74.4]	159 [45.2]	103 [29.3]			90 [73.2]	57 [46.3]	33 [26.8]		
BMI					0.919	0.338				/	/
	≤25	292 [83]	180 [51.1]	112 [31.8]			/	/	/		
	>25	60 [17]	33 [9.4]	27 [7.7]			/	/	/		
Tumor size (mm)					9.507	0.002*				1.225	0.268
	<50	182 [51.7]	96 [27.3]	86 [24.4]			14 [11.4]	7 [5.7]	7 [5.7]		
	≥50	170 [48.3]	117 [33.2]	53 [15.1]			109 [88.6]	71 [57.7]	38 [30.9]		
Tumor location					0.879	0.830				4.680	0.197
	Upper	105 [29.8]	67 [19]	38 [10.8]			39 [31.7]	21 [17.1]	18 [14.6]		
	Middle	60 [17]	34 [9.7]	26 [7.4]			37 [30.1]	24 [19.5]	13 [10.6]		
	Low	147 [41.8]	88 [25]	59 [16.8]			46 [37.4]	33 [26.8]	13 [10.6]		
	Overlap	40 [11.4]	24 [6.8]	16 [4.5]			1 [0.8]	0 [0]	1 [0.8]		

Differentiation					9.190	0.002*				4.508	0.034*
	Well/Moderately	133 [37.8]	67 [19]	66 [18.8]			28 [22.8]	13 [10.6]	15 [12.2]		
	Poor	219 [62.2]	146 [41.5]	73 [20.7]			95 [77.2]	65 [52.9]	30 [24.4]		
TNM stage					8.960	0.003*				5.547	0.019*
	I/II stage	131 [37.2]	66 [18.8]	65 [18.5]			54 [43.9]	28 [22.8]	26 [21.1]		
	III stage	221 [62.8]	147 [41.8]	74 [21]			69 [56.1]	50 [40.7]	19 [15.5]		

*$P < 0.05$ was considered significant

Supplementary Figure Legends

Figure S1. Flow diagram of the study. (A) Patient enrolment and study overview. **(B)** The expression of FABP family members in RNA-sequence. **(C)** IHC positive control for FABP from 3 cases of GC. Scale bars, 100 μm.

Figure S2. FABP4 expression and prognostic value in human GC. (A) Kaplan-Meier survival analysis of FABP4 expression in the internal cohort of patients with GC. The log-rank test was used to determine the *P* values. **(B)** Univariate and multivariate regression analyses were performed in the internal cohort (n = 352). **(C-D)** Kaplan-Meier and univariate and multivariate regression analyses were performed in the external cohort (n = 123).

Figure S3. Biological effects of FABP4 on GC cells *in vitro*. (A-C) Western blotting analysis of the protein levels of FABP4 and FABP5 in various GC cell lines and the construction of stably transfected GC cells. **(D-G)** The effects of FABP4 on the invasion, migration and adhesion of GC cells were detected by Transwell and adhesion assays. **(H-I)** Cell Counting Kit-8 was used to evaluate the effects of FABP4 on cell proliferation. **(J-K)** The effects of FABP4 on apoptosis of the cells were determined by flow cytometry.

Figure S4. Biological effects of FABP4 on GC cells *in vivo*. (A-F) The results obtained using a subcutaneous xenograft model of MGC-803 cells in BALB/c nude mice showed that FABP4 had no effect on the proliferation of GC cells *in vivo* (n = 3 for each mouse group). Tumour size was measured at indicated time points. Tumours were weighed after mice were sacrificed.

Figure S5. Verification of the relationship between FABP4 and CADM3 expression and evaluation of the function of CADM3 *in vitro*. (A) Both up-regulated and down-regulated candidate genes (n = 5) were selected for validation in public database TCGA and GSE15459. **(B)** CADM3 expression in various FABP4 groups of the external cohort was calculated. **(C)** Scatter plots showing the correlations between FABP4 and

CADM3 expression in the GSE15459 dataset. **(D)** Kaplan-Meier survival analysis of CADM3 expression in the internal cohort of patients with GC. **(E-F)** Detection of CADM3, CADM2 and CADM4 by western blotting after vector transfection. **(G-H)** Rescue experiment on the role of CADM3 in FABP4- associated metastasis showed that no significant difference in the invasive capacity of GC cells was found either when FABP4 was re-introduced with CADM3 knocked down or when FABP4 was disrupted with CADM3 overexpressed.

Figure S6. Analysis of the association between PPAR-γ and FABP4 in GC. (A) Potential protein-protein interactions of FABP4 were predicted using the STRING database. **(B)** Changes in PPAR-γ protein levels induced by various concentrations of rosiglitazone were assessed by western blotting. **(C)** Construction of the CADM3 promoter-luciferase reporter gene plasmid system.

Figure S7. Verification of the relationships between FABP4 and PPAR-γ by functional rescue assays. (A) The results of the Transwell assays showed that the effect of FABP4 overexpression on the migration and invasion of MGC-803 cells was reversed by PPAR-γ siRNA. **(B)** The results of the Transwell assays showed that the effect of FABP4 knockdown on the migration and invasion of MGC-803 cells was reversed by rosiglitazone (20 μM).

Figure S8. HDAC1-mediated chromatin inaccessibility reduces FABP4 expression in GC. (A) Analysis of FABP4 alterations in various types of GC. **(B)** Association between FABP4 DNA methylation and mRNA expression was analysed using cBioPortal. **(C)** Schematic diagram of the upstream regulatory mechanisms of FABP4. **(D)** Associations between HDAC1 and FABP4 and HDAC1 and CADM3 expression detected using the TCGA-STAD and GSE15459 datasets. **(E)** Association between HDAC1 and CADM3 IHC scores in two independent cohorts.
